# DRUGPATH – a novel bioinformatic approach identifies DNA-damage pathway as a regulator of size maintenance in human ESCs and iPSCs

**DOI:** 10.1038/s41598-018-37491-w

**Published:** 2019-02-13

**Authors:** Boris Kovacic, Margit Rosner, Karin Schlangen, Nina Kramer, Markus Hengstschläger

**Affiliations:** 1 Institute of Medical Genetics, Center for Pathobiochemistry and Genetics, Medical University of Vienna (MUV), Vienna, Austria; 2 Section for Biosimulation and Bioinformatics, Center for Medical Statistics, Informatics and Intelligent Systems, Medical University of Vienna (MUV), Vienna, Austria

## Abstract

Genetic and biochemical screening approaches often fail to identify functionally relevant pathway networks because many signaling proteins contribute to multiple gene ontology pathways. We developed a DRUGPATH-approach to predict pathway-interactomes from high-content drug screen data. DRUGPATH is based upon combining z-scores of effective inhibitors with their corresponding and validated targets. We test DRUGPATH by comparing homeostatic pathways in human embryonic stem cells (hESCs), human induced pluripotent stem cells (hiPSCs) and human amniotic fluid stem cells (hAFSCs). We show that hAFSCs utilize distinct interactomes compared to hESCs/hiPSCs and that pathways orchestrating cell cycle and apoptosis are strongly interconnected, while pathways regulating survival and size are not. Interestingly, hESCs/hiPSCs regulate their size by growing exact additional sizes during each cell cycle. Chemical and genetic perturbation studies show that this “adder-model” is dependent on the DNA-damage pathway. In the future, the DRUGPATH-approach may help to predict novel pathway interactomes from high-content drug screens.

## Introduction

Human ESCs have no expansion limit and represent a source for differentiated cells stemming from all three embryonic germ layers. Owing to these properties, hESCs hold a great promise for regenerative medicine. Whether or not regenerative medicine will become applicable in the future depends largely on our ability to derive and exploit ethically unproblematic cells with the closest possible characteristics to hESCs. These cells have become the golden standard for comparisons with *in-vitro* derived hiPSC. Therefore, knowledge about pathways they utilize to regulate the most essential homeostatic processes like survival, apoptosis, cell cycle and size are of utmost relevance.

So far, various approaches have been undertaken to identify molecular pathways that govern these processes. For instance, global transcription profiling qualifies for identification of genes which are up or downregulated between certain cellular states, but it is rather unsuitable for identification of steady-state pathway networks. In addition, many changes in transcriptional gene expression are rather consequences of unknown upstream signaling networks. Large scale RNAi-screens are more persuasive, however, they generate a large number of false-positives, yielding only few specific pathways^1–3^. Drug-screens are suitable for the analysis of homeostatic pathways because small-molecule inhibitors may act potently and instantaneously on their specific targets. However, intracellular networks are largely redundant, - i.e. many signaling proteins are shared between pathways - and thus, inhibition of one component will affect multiple pathways. Additionally, inhibitors have different specificities for on-targets and may also provoke off-target responses. Therefore, when screening, it is recommendable to use multiple inhibitors targeting the same on-target pathways. Comparable functional responses arising from multiple inhibitors targeting the same specific targets point to genuine on-target effects of the respective inhibitors. In contrast, distinct functional responses arising from inhibitors targeting the same specific targets may indicate that involvement of yet unknown pathways. In this study, we employ the power of bioinformatics to predict involvement of more distant pathways from the screening data. Generally spoken – very similar responses from inhibitors that are supposed to target the same pathway will minimize the number of outlier pathways. In contrast, different or even contrary responses of inhibitors targeting the same pathway will increase the amount of possibly involved pathways.

Comparisons between hESCs and hiPCSs have been undertaken to elucidate the mechanisms of pluripotency by functional screening, with major focus on cellular viability^4,5^ and differentiation^6–8^. Regulation of cell cycle, size and the balance between survival and apoptosis are highly evolutionary-conserved processes that need to be constantly maintained. So far, homeostatic mechanisms regulating cell cycle, size and viability have remained largely enigmatic in hESCs, hiPSCs and hAFSCs^9–12^.

In this study, we combine distinct bioinformatic methods to identify pathway interactomes that regulate cell cycle, size, survival and apoptosis of hESCs, hiPSCs and hAFSCs. Using the DRUGPATH-approach, we are able to predict pathway interactomes from hits which we obtained in a high-content inhibitor screen. We confirm the outcomes of our screen by validating previously published pathways (PI3K p110α, HDAC1/Notch1-axis) using chemical and genetic manipulation. Finally, we also identify a novel regulator of size in hESCs/hiPSCs - the ATM-signaling pathway.

## Results

### High-content screening to determine homeostatic mechanisms in distinct stem cells

We hypothesized that homeostatic processes may be differentially regulated in hESCs, hiPSCs and hAFSCs. To identify pathway networks, which regulate homeostatic processes, we measured changes in survival, apoptosis, cell cycle or size upon treatment with 81 selective small molecule inhibitors using flow cytometry. To this end, all cells have been stained intracellularly with propidium-iodide (PI) and information about ten different homeostatic parameters has been obtained simultaneously (Fig. S1, upper panel): (1–2) relative amount of living cells compared to untreated controls (relative survival) and their size, (3–4) proportion of apoptotic cells and their size, (5–10) three distinct cell cycle stages and their corresponding sizes. Notably, we have chosen one common inhibitor concentration (5 µM) for performing the screen. This is considerably lower than concentrations used in comparable previous screens^4,5^ and was aiming at reducing off-target effects. In most cases, we used two to four different inhibitors targeting the same pathway.

Initially, we considered that some inhibitors may enforce differentiation of human pluripotent stem cells (hPSCs). To avoid this unintentional effect, we sought to reduce duration of inhibitor treatments. This seems reasonable because previous work has shown absence of differentiation markers before day 3 of hESC-differentiation^13,14^. Thus, we initiated a pilot screen designed to measure the proportion of pluripotency markers in comparison to differentiation markers for ectoderm, mesoderm and endoderm. To exclude the possibility that this effect is induced by some inhibitors selectively killing pluripotent cells while leaving more differentiated cells unaffected, we included a marker for apoptosis in our pilot screen. We used 27 different inhibitors and set the length of treatments to 48 hours. Inhibitors inducing stem cell differentiation do not increase the proportion of apoptotic cells, but lose the expression of stem cell marker Oct4A, while gaining the expression of either ectodermal or mesodermal or endodermal markers (Fig. S2A,B). Importantly, we observed no evidence for differentiation under these conditions (Fig. S2C).

We have screened 9 different cell lines: 3 hiPSC lines (iPS-IMR90-1, iPS-Foreskin-2, iPS-DYR0100), 3 hAFSC lines (A1, H1, Q1) and 3 hESC lines (WA01, WA09, WA19) in technical triplicates. To avoid identification of unspecific pathways, we deployed distinct biological replicates and - where available - we used cell lines of distinct genetic backgrounds, tissue origins, reprogramming methods and sex (Table S1). To prevent detection of pathways resulting from a change in culturing method, we refrained from using density-type hPSC-cultures^4,5,15^ and developed a method allowing colony-type cultivation of hiPSCs and hESCs in a 96-well format (Materials & Methods).

### Developing the DRUGPATH-approach for comparative analysis

**Figure 1:**
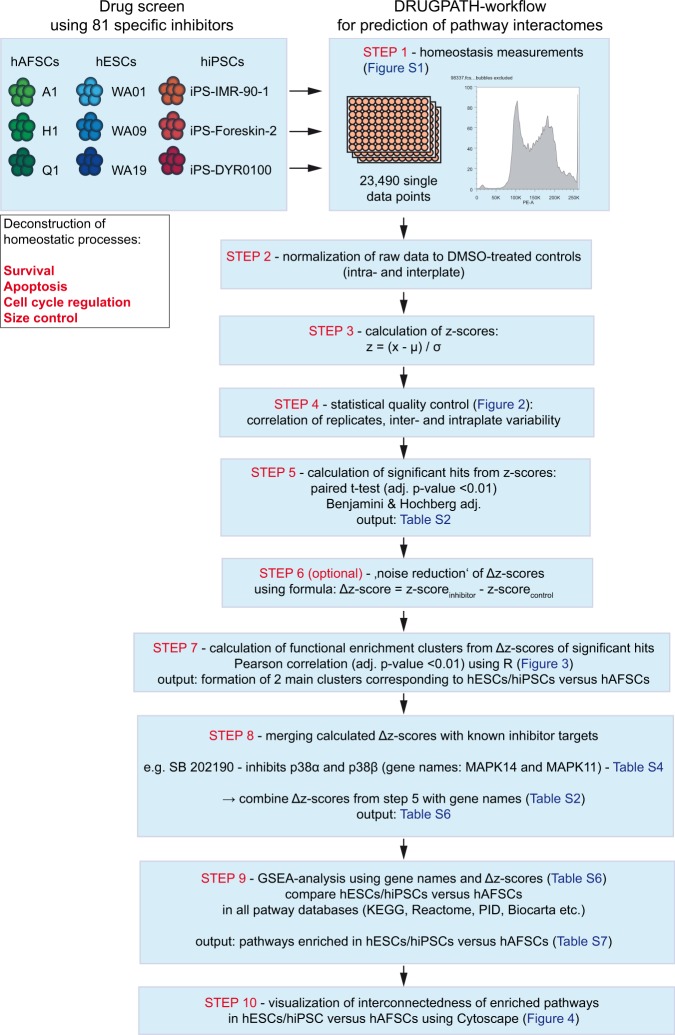
The DRUGPATH-workflow. DRUGPATH is a bioinformatic approach to process data from high-content drug-screens into pathway-interactomes containing vulnerable pathways. A high-content drug-screen has been performed aiming at comparing 81 drug-responses during 10 homeostatic processes in 3 different human stem cell populations. Steps 1 to 4 comprise of raw data processing, normalization, transformation into z-scores and statistical quality control testing, respectively. Significant inhibitor hits (adj. p < 0.01) are further subjected to functional enrichment analysis (clustering) to obtain information about similarity/difference among samples and among inhibitor-responses (steps 5–7). Notably, hESCs and hiPSCs constantly clustered together, while hAFSCs formed a separate cluster. In step 8, known target genes have been assigned to each effective inhibitor – thus, transforming all hits into lists comprising of gene names and corresponding calculated z-scores from our screen. Finally, the gene name/Δz-score-lists have been subjected to GSEA-analysis and pathway-interactome networks have been visualized from GSEA-enriched hits using the Cytoscape app (steps 9–10).

**Figure 2:**
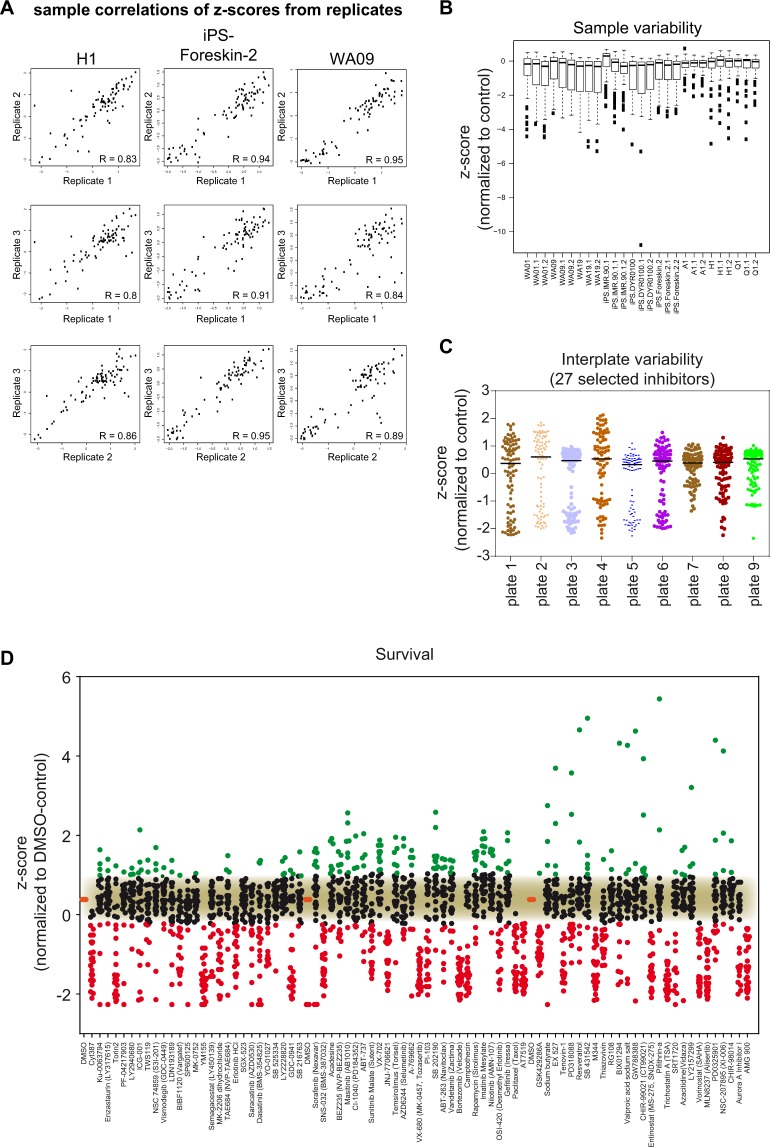
Statistical quality control of screen. Z-scores were used to calculate all measurements of statistical quality. (**A**) Low variance among replicates is depicted by representative scatterplots showing correlations of z-scores between all replicates for H1 cells, iPS-Foreskin-2 cells and WA09 cells. (**B**) Low Intraplate variability is represented by boxplots showing the sample variability for the ‘survival’ process. Boxplots represent medians ± SEM of all z-scores for each sample (i.e. cell line). (**C**) Scatterplots depicting the low interplate variability among all samples. Scatterplots show distribution of z-scores on 9 different 96-well plates containing the same cell line and the same collection of 27 inhibitors. Lines in scatterplots represent medians (**D**) Scatterplot depicting z-scores from all inhibitor responses and cell lines affecting the ‘survival’ process. Significant hits have been determined by student’s t-test (p < 0.01). Values above (green dots) or beneath (red dots) the noise-cutoff (black dots) represent significant hits.

We developed a bioinformatic workflow that we termed DRUGPATH - consisting of 10 steps, which ultimately lead to predictions of pathway-interactomes from drug-screening data (Fig. 1 and supplementary methods). In detail, we first normalized raw data values to internal controls that have been included into 96-well plates and then calculated z’-scores from all data (steps 1–3). Using z-scores over z’-factors is more advantageous because high signal to noise values are not transformed to ≤1, the formula allows negative z-scores and it does not generate zero values, which cannot be used during subsequent GSEA analysis. Z-scores were next used to perform statistical quality control of the screen (step 4). Indeed, there was a good correlation between all three technical replicates for each cell line (Fig. 2A). We could further exclude any possible effects caused by the intra-plate layout (i.e. distinct effects on margin wells; Fig. 2B) and confirm that the variance among different plates was sufficiently low (i.e. inter-plate alignment; Fig. 2C). All three analyses showed very good correlations – collectively suggesting that data from our screen may be used to reliably identify inhibitors involved in distinct homeostatic processes.

Next, we statistically discriminated between effective (Table S2) and non-effective inhibitors (Table S3) using paired t-test (adj. p < 0.01) and Benjamini & Hochberg adjustment (step 5). Notably, four inhibitors affecting survival and four inhibitors showing impact on apoptosis sampled out due to equal effects on all three hPSCs-groups (Table S3). Depending on possible outcomes of functional responses obtained in our screen, z-scores above (in case of apoptosis), or both, above and below noise, were regarded as hits (Figs 2D and S3). Of note, even though some cell lines exerted individual responses to some of the 81 inhibitors (Table S4), we have chosen to analyze common group-specific responses because those were likely to include shared pathway networks in hESCs, hiPSCs and hAFSCs. Notably, Δz-scores were created by subtracting control z-scores from all inhibitor z-scores (step 6).

**Figure 3:**
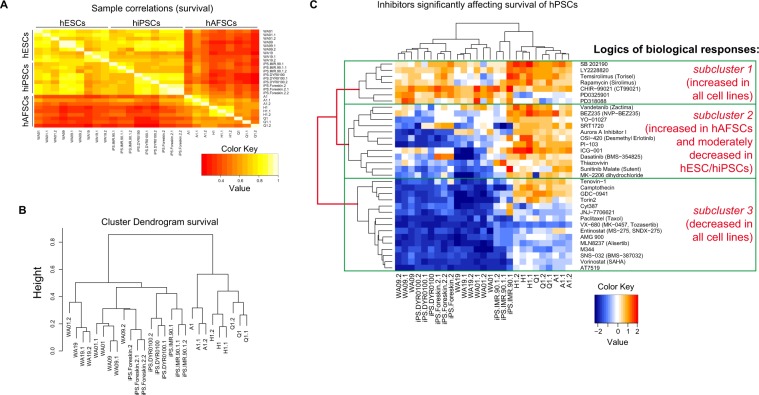
Unsupervised functional clustering identifies two main clusters corresponding to hESCs/hiPSCs versus hAFSCs. (**A**) Heatmap of Pearson correlations of all cell lines (samples) used in the screen for the ‘survival’ process. Notably, two large clusters comprising of hESCs/hiPSCs and hAFSCs separate from each other. (**B**) Dendrogram depicting Euclidian distances of all cell lines using Spearman correlations. Notably, there is a close distance between hESC and hiPSCs, as opposed to hAFSCs. (**C**) Heatmap showing unsupervised hierarchical clustering of all significant inhibitors affecting ‘survival’. Major subclusters arising by hierarchical clustering are depicted by green boxes.

We further performed sample clustering and hierarchical functional enrichment correlations (clustering) of all significant hits (Pearson-correlation, adj. p < 0.01; Fig. 3A–C). These analyses gave us hints about possible similarities among the samples (stem cell groups) and among inhibitor-responses (step 7). Interestingly, sample clustering of all biological processes constantly revealed outputs showing a high degree of similarity between hESCs and hiPSCs, as opposed to hAFSCs (Figs 3B and S4). There was no significant difference in the variability between hESC- and hiPSC-groups compared to differences among cell lines within each group. In contrast, a tight cluster comprising of hAFSC-samples separated from the other two cell groups (Figs 3C and S5). This was further substantiated by (unsupervised) hierarchical functional clustering (Fig. 3C and Figs S6–S9) using all cell lines and all effective inhibitors. All hierarchical heatmaps revealed separation of hAFSCs from hiPSCs/hESCs. In addition, we found 3 (to 4) subclusters revealing distinct inhibitor-responses and which can further be backtracked according to the logic of observed inhibitor responses, according to the mathematical relationship of relatedness (Pearson correlation) and according to the order of branching (i.e. 1^st^ and 2^nd^ branching). For example, inhibitors affecting “survival” (Fig. 3C) subdivide into 3 subgroups corresponding to (I) inhibitors increasing survival in all cell populations (positive z’-scores), (II) inhibitors increasing survival in hAFSCs and moderately decreasing survival in hiPSCs/hESCs and (III) and inhibitors decreasing survival in all cell lines (negative z-scores).

In order to predict homeostatic signaling pathways in stem cells, we next sought to transform information about effective inhibitors into information about proteins that are targeted by each inhibitor. To this end, we collected gene names of all validated targets (Table S4) for each effective inhibitor up to 5 µM concentrations (available at selleckchem.com). To seek for biologically-relevant pathways, we blasted gene names of our hits against DAVID (GO), REACTOME and STRING databases. Remarkably, we have yielded highly similar lists of enriched pathways from all three databases (Table S5) - suggesting a strong support for the validity of the proposed pathways. It is important to mention that from these results it was impossible to tell if the respective pathways are enriched in hESCs, hiPSCs, hAFSCs, or in all of them. Gene set enrichment analysis (GSEA) is a powerful bioinformatic tool that can calculate and compare enrichments of gene sets in two distinct cell populations, however GSEA requires gene names and relative expression values for computation. The high correlation of hESCs and hiPSCs in all biological processes has prompted us to consolidate these two groups into a ‘hESCs/hiPSCs-group’ for comparison with hAFSCs. Thus, we combined the gene names of validated inhibitor-targets (Table S4) with the z-scores obtained in our screen (Table S2) to yield hybrid gene-Δz-score lists (Table S6) that can be used to run GSEA (step 8 in Fig. 1). With these lists, we performed GSEA-analysis and looked specifically for enriched pathways (step 9 in Fig. 1) GSEA-hits (Table S7) were eventually subjected to pathway network reconstruction using the Cytoscape algorithm (step 10 in Fig. 1).

**Figure 4:**
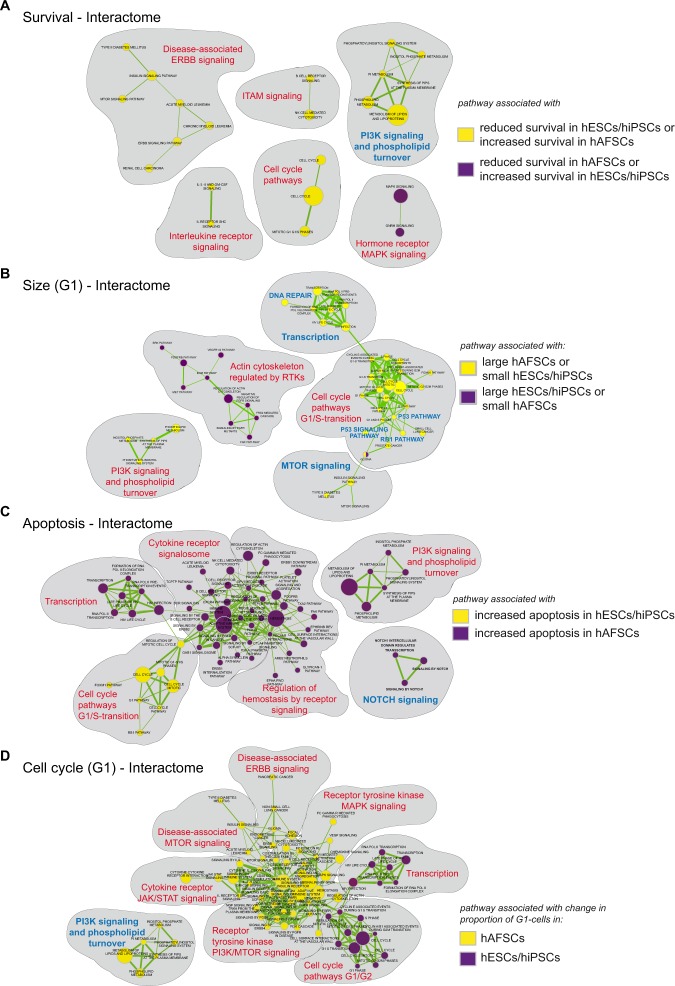
Modelling of pathway interactomes. (**A**) Landscape of interactomes regulating ‘survival’. Pathways associated with reduced survival in hESCs/hiPSCs (or increased survival in hAFSCs) are represented by yellow nodes. Pathways associated with reduced survival in hAFSCs (or increased survival in hESCs/hiPSCs) are colored in lilac. (**B**) Pathway interactomes of ‘size-regulation’ (during G1-phase). Pathways associated with large hAFSCs (or small hESCs/hiPSCs) are represented by yellow nodes. Pathways associated with large hESCs/hiPSCs (or small hAFSCs) are colored in lilac. (**C**) Pathway interactomes of ‘apoptosis’. Pathways associated with increased apoptosis in hESCs/hiPSCs are represented by yellow nodes. Pathways associated with increased apoptosis in hAFSCs are colored in lilac. Notably, a decrease in apoptotic cells has not been observed in our screen. (**D**) Landscape of interactomes regulating ‘cell cycle (G1-phase)’. Pathways associated with changes in the proportion of G1-cells in hAFSCs are represented by yellow nodes. Changes in the G1-proportion of hESCs/hiPSCs are colored in lilac. (**A**–**D**) The size of the nodes is proportional to the total number of genes within the pathway. The thickness of interactions is proportional to the number of shared genes (cutoff: 0.05).

The outcomes of the DRUGPATH-analysis for each homeostatic process are summarized in Fig. 4. Overall, the pathways differed in the way they fitted into larger landscapes: some landscapes showed a strong degree of interconnectivity resulting in fewer and larger interactomes, while others consisted of more and smaller interactomes. For instance, pathways orchestrating “survival” (Fig. 4A) and “size” (Fig. 4B) exerted low interconnectivity, while “apoptosis” (Fig. 4C) and “cell cycle” (Fig. 4D) revealed a strong interconnectivity of enriched pathways. Some interactomes (independent of size) consisted of pathways that were entirely associated to one certain cell group. We called these interactomes “group specific”. Interactomes displaying areas associated with distinct hPSC-groups were termed “universal”, because all groups are incorporated in one network landscape. The landscapes corresponding to “survival” and “size” were solely composed of “group-specific” interactomes. In contrast, >85% of apoptotic pathways were enriched in ‘universal interactomes’ (Fig. S10). Also during cell cycle progression, a clear majority of 72.4%, 74% and 92.3% of pathways resided in ‘universal interactomes’ in G1-, S- and G2/M-phases, respectively (Figs S10–S12).

### Validation of predicted interactomes

**Figure 5:**
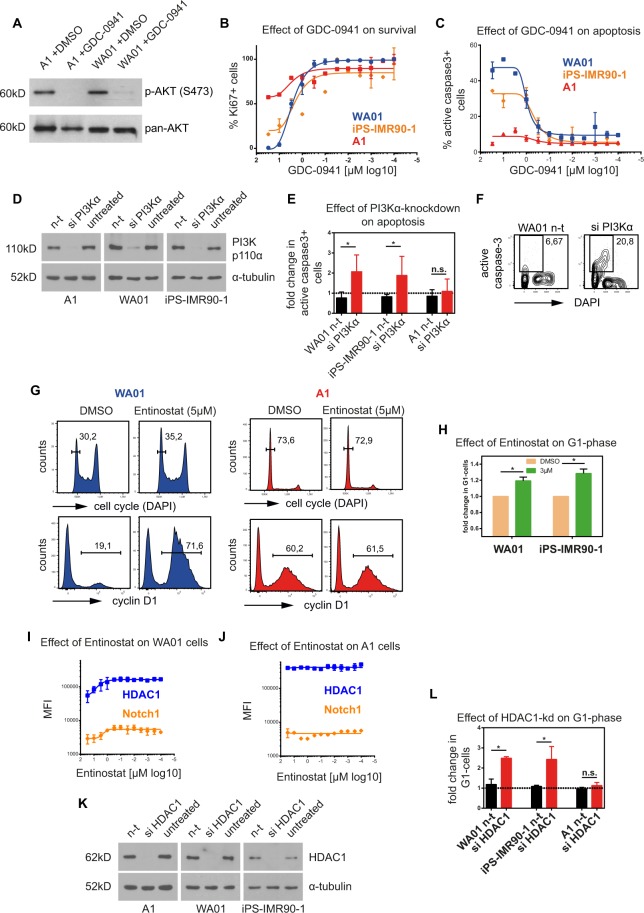
Validation of pathway interactomes predicted for ‘survival’ and ‘apoptosis’ processes. (**A**) GDC-0941-treatment inhibits downstream kinase AKT. Phosphorylation of AKT (S473) is inhibited after 4 h upon 5 µM GDC-0941-treament in A1 and WA01 cells. (**B**) Functional effect of GDC-0941-titrations on survival. Percentage of cells expressing the proliferation marker Ki67 decreases in a dose-responsive manner in WA01 and iPS-IMR90-1 cells after 24 h. (**C**) Functional effect of GDC-0941-titrations on apoptosis. Percentage of cells expressing active caspase-3+ is increased in WA01 and iPS-IMR90-1 cells in dose-responsive manner. (**D**) Validation of PI3Kα-knockdown efficiency in A1, WA01 and iPS-IMR90-1 cells. (**E**) Functional effect of PI3Kα-knockdown on apoptosis. Proportion of active caspase-3+ cells increased 2-fold upon PI3Kα-knockdown in WA01 and iPS-IMR90-1 cells. Bars represent means ± SEM of three independent experiments. (**F**) Representative FACS-plots showing percentage of active caspase-3+ WA01 cells upon PI3Kα-knockdown. (**G**) Cell cycle plots showing an increase in G1-phase in WA01, but not in A1 cells, after treatment with Entinostat (upper panels). FACS-histograms of CyclinD1 expression in WA01 (left) and A1 cells (right) before and after treatment with Entinostat (lower panels). The percentage of cyclinD1-expressing cells is increased in WA01 cells. (**H**) Fold increase in the percentage of G1-cells in WA01 and iPS-IMR90-1 cells upon 12 h Entinostat-treatment. (**I**,**J**) Notch1 and HDAC1-expression levels (MFI) in G1-cells correlate inversely with increasing Entinostat concentrations in WA01 cells (**I**), but not in A1 cells (**J**). (**K**) Validation of HDAC1-knockdown efficiency in A1, WA01 and iPS-IMR90-1 cells. (**L**) Functional effect of HDAC1-knockdown on G1-phase. Fold change in G1-cells is elevated upon HDAC1-knockdown in WA01 and iPS-IMR90-1 cells. Bars represent means ± SEM of three independent experiments. *n-t -* non-target siRNA control.

To validate proposed interactomes, we selected well-known pathways with differences between hESCs/hiPSCs and hAFSCs and performed further chemical and genetic perturbation studies. A network involving PI3K-signaling pathways was predicted to affect hESCs/hiPSCs during survival and during apoptosis (see Fig. 4A,C). GDC-0941 - a selective PI3Kα/δ inhibitor - resulted in the reduced phosphorylation of the downstream kinase AKT at S473 in both, hAFSCs and hESCs (Fig. 5A). Titrations of GDC-0941 (30 µM–300 pM) showed a reduction of Ki67+ cells in WA01 and iPS-IMR90-1 after 24 h in a dose-responsive manner, albeit not in A1 cells (Fig. 5B). To examine if GDC-0941 induced apoptosis in hESCs/hiPSCs, we measured expression of active caspase-3 upon GDC-0941-treatment. Titrations of GDC-0941 revealed an increase in active caspase-3+ WA01 and iPS-IMR90-1 cells in a dose-responsive manner after 12 h (Fig. 5C), confirming that inhibition of PI3Kα/δ-activity induced apoptosis of hESCs/hiPSCs. We next tested the possibility that A1 cells expressed lower levels of PI3K p110α and are therefore insensitive for GDC-0941-inhibition. A1, WA01 and iPS-IMR90-1 cells expressed similar PI3K p110α-levels under steady state conditions (Fig. S13). To corroborate under defined genetic conditions that PI3K p110α is essential to sustain survival of hESCs/hiPSCs, we performed knockdowns of PI3K p110α (Fig. 5D). We observed a prominent increase in the proportion of active caspase-3+ cells in WA01 and iPS-IMR90-1 cells, but not in A1 cells (Fig. 5E,F), suggesting that the survival of hESCs/hiPSCs, but not of hAFSCs is dependent on PI3K p110α.

To confirm DRUGPATH-predictions from G1-interactomes, we validated the relevance of Notch-pathway in distinct cell groups. Notch-signaling has been implicated in G1/S-delay and acceleration of neuronal differentiation of neural stem cells^16^. HDACs have been shown to regulate the signal transduction downstream of Notch-signaling^17^. Here, short treatment (4 h) with selective HDAC1/3-inhibitor Entinostat strongly influenced the expression of cyclinD1 in WA01, but not in A1 cells (Fig. 5G). Importantly, Entinostat did not increase cyclinD1-expression levels in G1-cells, but rather increased the proportion of cells expressing cyclinD1 (Fig. 5G), suggesting that more hESCs entered G1. Accordingly, longer treatments (12 h) of WA01 and iPS-IMR90-1 cells with Entinostat increased the proportion of G1-cells (Fig. 5H). Titrations of Entinostat as well as another HDAC-inhibitor, Trichostatin A (TSA), revealed a reduction of Notch1- and HDAC1-levels in WA01 cells in a dose responsive manner (Figs 5I,J and S14A). Steady-state HDAC1-levels were largely comparable between A1, WA01 and iPS-IMR-90-1 cells (Fig. S13). Efficient HDAC1-knockdown in these cells (Fig. 5K) led to a 2-fold increase in the proportion of G1-cells in WA01 and iPS-IMR90-1 cells, but not in A1 cells (Fig. 5L). Notably, prolonged treatments (36 h) with HDAC-inhibitors did not result in differentiation, but in an increase in apoptosis of hiPSCs/hESCs (data not shown). Together, we conclude that in hESCs and hiPSCs the HDAC/Notch-axis primarily regulates G1-length.

### ATM is a novel regulator of size maintenance in hESCs/hiPSCs

**Figure 6:**
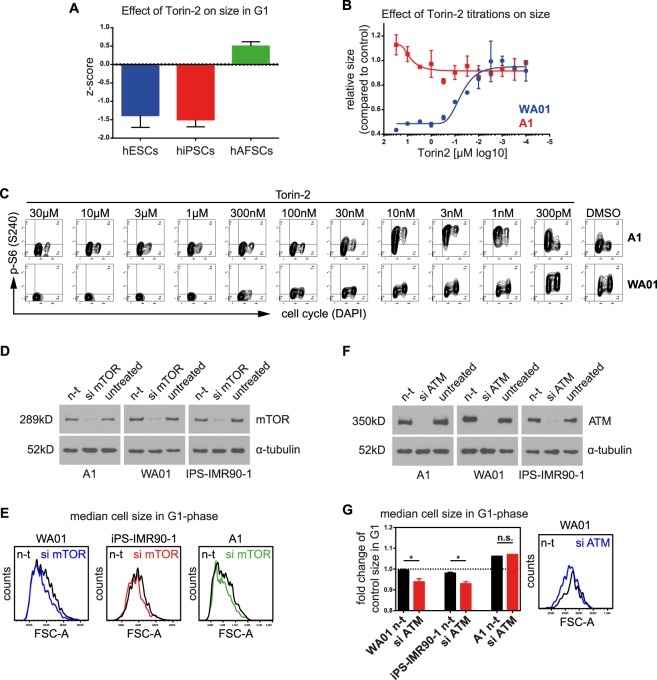
Pharmacological inhibition or knock-down of ATM reduces cell size in hESCs/hiPSCs. (**A**) Average effect of Torin-2 on hPSC-groups. Note that in contrast to other mTOR-inhibitors (Fig. S15), Torin-2 exerts similar negative size effects on both, hESCs and hiPSCs. (**B**) Functional effect of Torin-2-titrations on size. Torin-2 recapitulates the negative size-effects in a dose-responsive manner in WA01 cells, but not in A1 cells. (**C**) Molecular effect of Torin-2-titrations. mTOR-dependent phosphorylation of S6 (S240) is strongly diminished upon increasing Torin-2 concentrations in both, WA01 and A1 cells. (**D**) Validation of mTOR-knockdown efficiency in A1, WA01 and iPS-IMR90-1 cells. (**E**) Effect of mTOR-knockdowns on size of hPSCs. mTOR-knockdowns reduced the average G1-size of all hPSCs - ruling out that mTOR alone is sufficient to explain different size mechanisms in hESCs/hiPSCs versus hAFSCs. (**F**) Validation of ATM-knockdown efficiency in A1, WA01 and iPS-IMR90-1 cells. (**G**) Effect of ATM-knockdowns on size of hPSCs. Knockdown of ATM reduced the average G1-size in WA01 and iPS-IMR90-1 cells, but not in A1. Bars represent means ± SEM of four independent experiments. One representative histogram of WA01 cells showing FSC-A-changes upon ATM-knockdown is depicted on the right. *n-t* - non-target siRNA control.

To confirm DRUGPATH-predictions from the “size” interactome, we validated the role of mTOR-pathways during size-maintenance (see Fig. 4B). In total, 4 different mTOR-inhibitors have been retrieved as size modulators in the screen (Torin-2, Rapamycin, Temsirolimus and BEZ-235) – albeit with opposing effects on size: Rapamycin recapitulated recently observed size reduction^18^ in hAFSCs (Fig. S15A–C), however, Torin-2 reduced the size of hESCs/hiPSCs but increased the size of hAFSCs (Fig. 6A). To validate this effect, we volumetrically measured cell size in response to various titrations of Torin-2 using the Casy cell counter. WA01 cells indeed responded with size-reduction in a dose-dependent manner, while A1 cells did not (Fig. 6B). Importantly, Torin-2 efficiently inhibited its major on-target - p-S6 (S240) - in both, WA01 and A1 cells at similar doses although with distinct kinetics (Fig. 6C). In WA01 cells, p-S6 (S240) levels in G1 were more sensitive to Torin-2-inhibition than p-S6 (S240) levels in G2-phase (Fig. 6C). Because hAFSCs expressed similar mTOR-levels compared to hESCs/hiPSCs (Fig. S13) and responded with comparable size reduction upon mTOR-knockdowns (Fig. 6D,E), we hypothesized that the observed size-modulating effect of Torin-2 may not act via mTOR-inhibition.

Torin-2 is a second generation ATP-competitive inhibitor exerting an increased potency against the serine/threonine kinase ATM^19^, which has been implicated in regulation of DNA-damage induced stress by p53-signaling in hPSCs^7^. As DNA-damage/transcription has been predicted by DRUGPATH to have a role in “size”-interactome (see Fig. 4B), we tested the possibility that ATM, as a regulator of DNA-damage signaling, may influence the size of hESCs/hiPSCs - as opposed to hAFSCs. All hPSCs expressed comparable ATM-levels under steady-state conditions (Fig. S13), but remarkably, efficient ATM-knockdowns (Fig. 6F) reduced the size of WA01 and iPS-IM90-1 cells, while no significant size changes were observable in A1 cells (Fig. 6G). Our data reveal that, as opposed to mTOR-signaling, ATM-signaling may maintain the regular size of hESCs/hiPSCs.

### Differences in general strategies of size control

**Figure 7:**
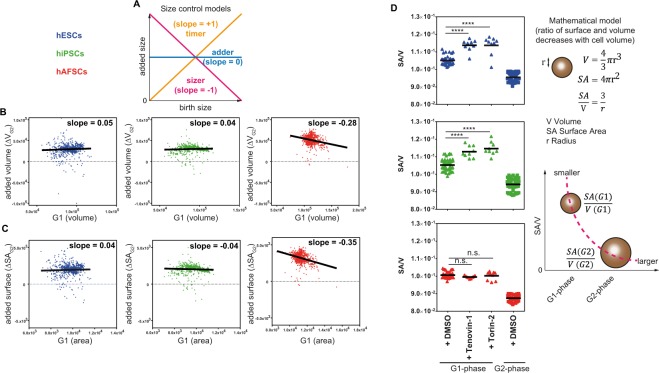
“Adder-model” in hESCs/hiPSCs is dependent on the DNA-damage pathway. (**A**) Predictions from size control models as suggested by Jun *et al*.^21^. Slope +1 corresponds to a perfect ‘timer’, 0 to a perfect ‘adder’ and −1 to a perfect ‘sizer’. (**B**) Relationship between ‘birth size’ and ‘added size’ (ΔV_G2_) is shown using all inhibitor-responses to each hPSC-group (n = 783 each). (**C**) Relationship between ‘birth surface area’ and ‘added area’ (ΔSA_G2_) is depicted for each hPSC-group (n = 783 each). (**B**,**C**) Note that hESCs and hiPSCs are nearly perfect ‘adders’, while hAFSCs represent moderate ‘sizers’. (**D**) Mathematical model for a mechanism that senses the ratio between surface area and volume (Harris *et al*.^23^) has been adapted to conditions used in our screen (spherical cells – see results). According to this model - while cells increase their volume during cell cycle progression, the relative rates of surface area to volume decrease between G1 and G2. Conversely, smaller cells must have increased surface area to volume ratios. To test whether the adder model is dependent on the DNA-damage pathway, we treated hESCs, hiPSCs and hAFSCs with inhibitors targeting Sirtuins and ATM (Tenovin-1 and Torin-2). As predicted from the mathematical model depicted in the right panel, treatment with Torin-2 and Tenovin-1 increased the SA/V-ratio in hESCs and hiPSCs, but not in hAFSCs (p < 0.0001).

We next addressed if hESCs, hiPSCs and hAFSCs follow distinct general strategies of size control. Previous studies have suggested that cells can control their size either by measuring time, absolute size or relative size – resulting in “timer-”, “sizer-” or “adder-” models, respectively^20,21^. A positive slope in volume-increase indicates a “timer”, a negative slope correlates to a “sizer”, while a slope equaling zero indicates a “constant adder” (Fig. 7A). We addressed which of these size models applied to hESCs, hiPSCs and hAFSCs. Facs-analysis using PI allowed for simultaneous measurements of ten thousand cells per well under various inhibitor-treated or control conditions. Thus, for each condition, we can extract size values (FSC-A) of cells residing in G1- and G2-phases, respectively. Next, to analyze if the stem cells grew regularly during cell cycle, we checked if the overall sizes in G1 correlated to the respective sizes in G2 with a strong positive slope (approx. +1). As expected, all cell groups showed a strong positive correlation between ‘birth size’ (G1) and ‘size at division’ (G2), suggesting a “normal” doubling of the volume during cell cycle progression (Fig. S15D).

As suggested by previous studies, a clear separation between the models is possible if relative volume-increases (“added volume”) are correlated to corresponding initial sizes in G1 (“size at birth”)^20–22^. Therefore, we calculated ΔV-values (V_G2_ − V_G1_ = ‘added size’) from the screen data and correlated them to the corresponding “sizes at birth” (G1). Interestingly, the size checkpoints were differentially regulated in hESCs/hiPSCs and hAFSCs: hESCs/hiPSCs follow the ‘adder’-model by growing equal volumes and equal surfaces before division irrespective of the ‘birth size’. In contrast, hAFSCs showed a negative slope and thus obey to the ‘sizer’-model (Fig. 7B,C). Furthermore, we found that the Gaussian distribution (variance) of all added sizes in hAFSCs is much higher compared to hESCs or hiPSCs (Fig. S15E) – which is also compatible with the sizer model in which the cells grow different volumes between G1 and G2 in order to end up with one constant size at the end of G2.

Validation of predictions from size-interactomes indicated that ATM is a regulator of size-maintenance in hESCs/hiPSCs, but not in hAFSCs (Fig. 6). As hESCs/hiPSCs seem to utilize the “adder model” for size control, we next addressed the question whether the DNA-damage-pathway (i.e. ATM) may have a role in regulation of the ‘adder’-model in hESCs/hiPSCs. Recent work has provided mathematical framework to the understanding of the ‘adder model’ by showing that cells can sense accumulation of cell surface material that is necessary to synthetize two new membrane caps^23^. Depending on the nutrients and on the time they need to fulfill this synthesis, cells may change the relative ratio of surface area (SA) to volume (V) during cell cycle – thereby becoming smaller or larger depending on culture conditions. Notably, this model has only been shown for bacteria. Our prediction for the stem cells was that hESCs/hiPSCs with blocked DNA-damage signaling would reduce their size and accordingly increase their SA/V-ratio. We treated distinct cell groups with Tenovin-1 and Torin-2, which specifically inhibit two components from different DNA-damage-pathways, Sirtuin1/2 and ATM, respectively. The relative contribution of Sirtuins to different DNA-damage pathways is not yet clear^24^. We calculated the SA/V-values using the mathematical assumption that cells resemble a spherical ball (microscopically confirmed in suspensions used for FACS). Indeed, treatment with both inhibitors increased the SA/V-ratios during the G1-phase specifically in hESCs/hiPSCs (Fig. 7D), as suggested by our model. Together, these data implicate two distinct DNA-damage pathways in the regulation of the “adder”-model in hESCs/hiPSCs.

## Discussion

Here we used a combination of bioinformatic methods termed DRUGPATH to predict homeostatic pathway interactomes from data obtained in an inhibitor screen using distinct human stem cell groups. We showed that hESCs and hiPSCs – as compared to hAFSCs - utilize largely different interactomes to control for cell cycle, size and survival. We validated our predictions using chemical and genetic perturbation studies and verified some previously reported (PI3K p110α, Notch1/HDAC1-axis, mTOR). Ultimately, we have identified a novel role for serine-threonine kinase ATM in size-maintenance of hESCs and hiPSCs.

Our bioinformatic approach has clear advantages over previous screens. Previous studies have analyzed genetic variabilities and differences in mRNA-transcripts and epigenetic marks^9–12,25–27^. We sought to identify essential pathways by screening for functional differences between the stem cell groups. This is highlighted by the total number of pathways identified in this study compared with previous screening data^1,4^ on which our methodology has been subsequently applied (Fig. S16). In our view, there are two major reasons for this: First, we used a limited number of inhibitors with high selectivity for specific targets, while others used large-quantity libraries with unknown selectivity. Second, the amount of measured variables in a high-content screen is likely to generate more identifiable pathways. This is exemplified by the highest overlap to our study when comparing data generated using a similar multiparametric flow cytometric screen^1^.

The validity of our predictions is highlighted by confirmations using chemical and genetic ablation studies. For validation, we have chosen hits that had previously been known to regulate survival and apoptosis in hPSCs. First, we confirmed the essential role of PI3K p110α for survival and differentiation of hESCs and hiPSCs^28,29^. In our hands, PI3Kα-inhibition using GDC-0941 inhibitor or PI3Kα-knockdown constantly produced lethal phenotypes without any effects on differentiation (Figs 2 and 5, Fig. S2 and data not shown). When chemically inhibited or genetically ablated from hESCs and hiPSCs, the lack of PI3Kα provoked apoptosis and cell death within approximately 4 h. One explanation for this observation is that GDC-0941 exerts a > 100-fold selectivity over LY294002, which has been used in previous studies^29,30^. Alternatively, it is possible that additional differentiation signals are needed, which are present in conditioned media used by others^28–30^. However, PI3K-signaling seems to be less critical for the survival of hAFSCs. Thus, the vulnerability towards PI3K p110α inhibition represents a cell intrinsic difference in survival signals between hESCs/hiPSCs and hAFSCs.

Second, we verified the role for HDAC1/Notch1-axis in the regulation of G1-phase of hESCs and hiPSCs. In contrast, this signaling pathway is presumably dispensable during G1 in hAFSCs. In line with this, HDAC-inhibition by two different inhibitors as well as HDAC1-knockdowns both resulted in selective G1-perturbation in hESCs and hiPSCs. The effect was accompanied by downregulation of Notch1-expression during G1-phase. This is in line with previous studies showing that Notch-inhibition delays G1/S-transition and increases G1-cell proportion^16^. Our data add to these findings that HDAC1-signaling downstream of Notch1 may be responsible for this effect.

In contrast to hiPSCs, hAFSCs are a naturally occurring and isolatable fetal cell population. Initially, hAFSCs have been attributed as ‘broadly multipotent’ because of distinct marker expression compared to hESCs^31^ or as ‘loosely pluripotent’^32^ because of their ability to differentiate into all three germ layers. hAFSCs are clearly distinct from hESCs and hiPSCs – however – this difference has not yet been addressed in detail. Here, we hypothesized that homeostatic processes like cell cycle, size and survival, may be differentially regulated in hESCs, hiPSCs and hAFSCs. Interestingly, the different characteristics of hAFSCs may be reflected in distinct pathway interactomes. Hence, hAFSCs may exhibit distinct pluripotency marker expression than hESCs/hiPSCs as a consequence of distinct pathway interactomes regulating their homeostatic processes.

The regulation of size has to our knowledge not yet been addressed in hPSCs. Here, we show that the maintenance of size in hESCs and hiPSCs is regulated by the ATM-signaling (Fig. 6). To the best of our knowledge, this is the first functional description of the serine/threonine kinase ATM in regulating size. Interestingly, this finding is supported by observations showing that ATM is a DNA-repair and cell cycle checkpoint regulator and that ATM-deficient mice have growth-retardation^33^. Hence, findings on ATM presented in this study represent a functional link between the cell cycle and size checkpoints in hPSCs.

In addition, we have identified the hESCs and hiPSCs utilize an unorthodox general mechanism of size control – the ‘size-adder’-model. Here, the cells grow exactly the same proportion of volume during one cell cycle. So far, the ‘adder’-model has only been shown to apply for bacteria – therefore, it is tempting to suggest that hESCs/hiPSCs may utilize a more rudimental program of size control than other differentiated mammalian cells. Interestingly, we also show that DNA-damage pathways may have a role in regulating the ‘adder-model’ in hESCs/hiPSCs. Indeed, when this pathway is inhibited the cells increase their cell surface to volume ratio in G1 (Fig. 7). By doing so, it is possible that hESCs/hiPSCs need more time to catch up with the regular volume and thus may prolong the cell cycle. One possibility why stem cells could have adapted this mechanism is that precise surveillance of DNA-errors by the DNA-damage pathway is vital for hESCs and hiPSCs. Thus, they may extend their cell cycle in order to gain more time to correct the DNA-errors. Our findings suggest that Sirtiun1/2 and ATM could have an important role in the regulation of this mechanism. So far, it is not clear if sirtuins and ATM act together to coordinate this function and how a molecular mechanism of interaction may look alike. Future studies shall clarify if binding of sirtuins to DNA is required for a proper function of ATM or whether both pathways may act independently to regulate the adder-model.

We anticipate that the DRUGPATH-approach and the pathway interactomes presented here may also provide a resource for disease-modelling using reprogrammed patient-derived hiPSCs. In addition, DRUGPATH-approach may be applied for all inhibitor-based screens and used to discover new mechanisms of action and find vulnerable pathways for future treatment strategies.

## Materials and Methods

### Principles of DRUGPATH-analysis and experimental design

#### Principles

on functional and molecular level, hESCs, hiPSCs and hAFSCs share some similarities (differentiation into all 3 germ layers, Oct3/4-expression etc.) and some differences (lack of some pluripotency markers, poised spontaneous differentiation into specific lineages, distinct morphologies, distinct epigenetic states etc.). In this study we assumed that these differences may arise from distinct programs (i.e. large protein networks), which distinct stem cells utilize to maintain their homeostatic processes like regulation of survival, cell cycle and size. Previous work has suggested that half-lives of proteins regulating homeostasis can be several magnitudes longer than a mammalian cell cycle and that – in general – mRNA abundance is not well correlated with protein abundance^34,35^. These findings indicated that analysis of global transcriptional or epigenetic states or screening by RNA-interference may be less productive than drug-screening. To identify pathways that are essential to control survival, apoptosis, cell cycle progression or size, we utilized 81 inhibitors specific for key signaling proteins. Since each of the 81 inhibitors has known target(s), we could deduce information about all validated targets and combine them with the magnitude of response measured in our screen. Using this information, we were able to bioinformatically calculate pathways that may have been affected by specific inhibitor hits.

#### Experimental design

Usually, outputs generated by drug-screens are lists of candidate inhibitors with statistically significant effectivity to a specific biological response in certain cell groups. These inhibitors need to be further validated in order to confirm their action. However, inhibitors have many targets and each target has many roles in different signaling pathways. Thus, to predict pathways that have directly been influenced by effective inhibitors, we developed the DRUGPATH-approach. The DRUGPATH-approach uses two variables from a drug-screen – the effective inhibitor (hit) gene name and the strength of its effectivity in the screen. To make this effectivity measure a useful variable, we first calculated z-scores (as opposed to z’-factors, which yield amplitudes between 0 < z′ < 1) for each condition and process applied in the screen. The advantage of z-scores is that they do not reduce relative amplitude of specific responses above noise and that they do not generate 0 values, which are useless in GSEA. In detail, we followed these principles for DRUGPATH-analysis (Fig. 1): steps 1–6 – raw data normalization, z-score and statistical quality control, calculation of significant hits (Table S2) and noise reduction (optional); step 7 – functional enrichment analysis (clustering) is necessary to reveal similarities and differences between compared groups. Our inhibitor screen constantly yielded high similarity between inhibitor-responses in hESCs and hiPSCs versus hAFSCs. Thus, to increase the possibility of identifying more conserved pathway networks, we further optimized our working hypothesis by asking for relevant pathways between hESCs/hiPSCs versus hAFSCs. A key step in the DRUGPATH-workflow is the consolidation of significant inhibitor hits and amplitudes of responses with target gene lists (step 8). Given that inhibitor X has one or multiple known target/s up to a working concentration of 5 µM, we can postulate that inhibition of at least one of these targets (or all possible) may have caused the observed phenotype (Table S7). Using multiple selective inhibitors we can statistically enrich the probability of predicting the relevant target or pathway. This has been done in step 9 using the GSEA-algorithm by specifically comparing hESCs/hiPSCs and hAFSCs against Molecular Signatures Databases v5.0 (http://software.broadinstitute.org/gsea/msigdb): C2: KEGG and Reactome as well as C5: BP (biological process), CC (cellular component) and MF (molecular function). Finally, visualization of nodes and connections has been performed using the Cytoscape app (step 10).

### Screen analysis

Facs data have been pre-analyzed using Facs Diva Software and exported to FlowJo for further analysis. Each sample has been checked individually and gatings adjusted if necessary (Fig. S1, lower panel). The resulting data compendium has been exported to Excel to perform normalization to internal controls (untreated, DMSO-treated). No differences between untreated and DMSO-treated cells have been observed for any parameter measured in this study.

### z-score calculations

We performed three parallel approaches for further statistical analysis: normalized raw values, log2-tranformed raw values and z-scores. Z-scores were calculated from each value using following formula: z = (x − μ)/σ, where x represents a normalized raw value of each cellular process (e.g. relative survival, % apoptotic cells etc.), µ is the mean of all values in a certain cellular process and σ is the standard deviation of all values of a certain cellular process.

### Statistical Analysis of hESCs, hiPSCs and hAFSCs

The entire analysis was done within R statistical scripting language environment (v.3.2). For comparisons between hPSC-groups, z-scores values, log2-values and raw values were considered for calculation using R’s ‘pairwise.t.test’ function. Benjamini & Hochberg adjustment method was applied to the p-values for multiple testing corrections. In addition, all comparisons have been calculated in GraphPad Prism 6. Normal data distribution has been tested using Kolmogorov-Smirnov test and calculated using One-way Anova and Dunnett’s multiple comparison post-test. All comparisons yielded >95% identical hits (data not shown). P < 0.01 (alpha = 1%) was accepted as statistically significant.

### Functional enrichment analysis

In order to visualize the correlations between the z-score values of the different samples, heatmaps of the matrix of Pearson correlations between the samples were plotted using the ‘heatmap.2’ function from the gplots package. Furthermore, heatmaps of inhibitor- z-scores with significant differences in means between the different groups (adj. p-value < 0.01) were plotted. Statistics were calculated using the ‘pairwise.t.test’ as described above. Hierarchical cluster analysis was performed using R’s ‘hclust’ function using the Euclidean distance matrices and a complete linkage clustering of Spearman correlation values of the inhibitor z-scores between the samples. Principal component analysis (PCA) was performed with the transposed z-scores using the R function ‘prcomp’, and the first two principal components were plotted. A full script can be obtained from here: https://github.com/karinschlangen/stemCells_pathways

### Superior network analysis

For superior-network analysis, we separately considered all significant inhibitors of survival, apoptosis, G1-phase, S-Phase, G2/M-phase, size of G1-phase cells, size of S-phase cells, size of G2/M-phase cells, size of apoptotic cells and size of all cells. We hypothesized that inhibitor-groups showing similar effects on hPSC-groups belong to common pathway networks utilizing common core proteins. Similar effectors have been consolidated into subclusters, yielding a total of 29 subclusters for all interrogated biological processes. Lists with significant inhibitors have been transformed into gene lists containing all published inhibitor targets effective at <5 µM concentrations (Table S7). To identify superior networks, we employed two strategies: First, gene ontology analysis was performed with (i) DAVID functional annotation tool (david.abcc.ncifcrf.gov) for biological processes and functional clusters, (ii) REACTOME pathway analysis (reactome.org) for statistically overrepresented pathways and (iii) STRING protein network interaction analysis (string.embl.de). The resulting outputs contained up to several hundred significant GO-terms enriched in each subcluster (Table S5) and did not differentiate between the hPSC-groups. Second, a parallel approach has been undertaken, which was primarily independent of subclusters, but dependent on enrichment to hPSC-groups. Therefore, we used gene lists containing significant targets and their respective fold-changes of z-scores (Table S6; compared to controls) to search for pathways enriched in hESCs/hiPSCs versus hAFSCs by gene set enrichment analysis (GSEA). GSEA, v2.2.1 was performed against Molecular Signatures Databases v5.0 (http://software.broadinstitute.org/gsea/msigdb): C2: KEGG and Reactome as well as C5: BP (biological process), CC (cellular component) and MF (molecular function). Analysis was performed with 1000 permutations on pre-ranked gene lists using the fold changes of the respective comparisons.

Significantly affected pathways/GO terms (Table S7; FDR < 0.25) were displayed using Cytoscape (cytoscape.org) and the Enrichment Map plugin. Enriched pathways are represented by nodes, which are grouped according to gene similarity within each pathway/GO term. The size of the nodes is proportional to the total number of genes within each pathway, while the width of the edges is proportional to the gene overlap between the pathways (number of shared genes), calculated using the overlap coefficient (cutoff: 0.05).

## Supplementary information

Supplementary Methods, Figures S1–18, Legends

Table S1

Table S2

Table S3

Table S4

Table S5

Table S6

Table S7

## Data Availability

Any data in this manuscript will be provided upon request.
