# Supplementary material for: DRUGPATH – a novel bioinformatic approach identifies DNA-damage pathway as a regulator of size maintenance in human ESCs and iPSCs
Source: Sci Rep. 2019 Feb 13;9:1897. doi: 10.1038/s41598-018-37491-w (PMC6374489; doi:10.1038/s41598-018-37491-w)
Supplement: Supplementary file 1 — Supplementary Methods, Figures S1–18, Legends [file 41598_2018_37491_MOESM1_ESM.pdf]

DRUGPATH – a novel bioinformatic approach identifies DNA-damage pathway as a regulator of size maintenance in human ESCs and iPSCs

Boris Kovacic, Margit Rosner, Karin Schlangen, Nina Kramer and Markus Hengstschläger

<u>Index:</u>

Supplementary Methods and References

Supplementary Figures 1-18

Supplementary Figure Legends

Supplementary Table Legends

Supplementary Methods

Cell culture

hESC lines and hiPSC lines were cultured in mTeSR1 (Stemcell Technologies) using a colony-type and feeder-free protocol. iPS-DYR0100 cells (ATCC-DYR0100 human induced pluripotent stem cells) were purchased form ATCC, while all other hiPSCs and hESCs were purchased from WiCell. The plates were pre-coated with Matrigel (Corning) in a medium containing DMEM/F12 (Invitrogen). The cells were fed daily and passaged when the colonies have reached a certain size (mid-stage colonies, every 3-4 days) using ReLeSR (Stemcell Technologies). hAFSCs were cultured as described previously[1] and passaged every 2-3 days using Trypsin/EDTA for 5 minutes.

Differentiation of hESCs has been performed by switching from mTeSR1 to TeSR-E6 medium (Stemcell Technologies) that does not contain TGFβ and bFGF and that has been optimized for differentiation protocols, screening assays and other settings where presence of TGFβ and bFGF is not desired. Differentiation time was between 5 and 6 days.

Screen

Logarithmically growing hPSCs were passaged and plated in 96-wells. To avoid detection of pathways resulting from the mere adaption of hESCs and hiPSCs to small-format wells usually utilized for high-throughput and high-content screening, we developed a method for hESCs and hiPSCs cultivation in 96-well format. In brief, hPSC-colonies were dissociated using ReleSR and plated on Matrigel-coated 96-wells using wide-bore 200 µl filter tips. The optimal splitting ratio has been determined to equal a ratio of one mid-stage 6-well to one 96-well plate (96-wells).

hAFSCs were splitted using Trypsin/EDTA, counted using Casy Counter (Roche) and plated at a density of 10,000 cells/well in a 96-well plate. Inhibitor-groups have been chosen to be selective for various targets involved in stem cell research. All inhibiotrs were purchased from Selleckchem (www.selleckchem.com). Compounds from the 'FDA-approved and late-stage in-trials'-group have already been used previously[2]. All inhibitors (Selleckchem, 10 mM stocks) have been prediluted at once to a 5 µM working concentration in the respective media and frozen as ready-to-use masterplates at -80 °C. All masterplates included inhibitors and controls in triplicates. Untreated and DMSO-treated conditions were used as controls. At screening, the growth medium of an hPSC line in 96-well format was exchanged with the content of the masterplate. After 48 h at 37 °C/5 % CO_2, the plates were immediately processed as follows: medium containing inhibitors was discarded and the remaining cells were washed with PBS (w/o Ca^{2+}/Mg^{2+}). Cells were detached using 40 µl Accutase (hESCs and hiPSCs) or 40 µl Trypsin/EDTA (hAFSCs) for 5 minutes. Detached cells were resuspended in 160 µl PBS or PBS/10%FCS, pipetted into V-bottom 96-wells and centrifuged at 400 g. After discarding the supernatant, the cells were resuspended in 4 % PFA (EMS) for fixation. The average time for procession of one plate was 4 minutes and 8 minutes/3 plates. Cells fixed in 4 % PFA were processes by centrifugation at 400 g for 5 min, resuspended in ice-cold methanol and frozen at -80 °C.

For measurement, fixed cells were centrifuged at 800 g, washed with PBS and resuspended in 150 µl propidium iodide containing RNase A (Becton Dickinson). The plates were incubated at 37 °C for at least 30 minutes and measurements were performed using a FACS Canto II (BD) equipped with 3 lasers and a high-throughput

sampler unit or using the Cytoflex S (Beckman Coulter) equipped with 4 lasers and a plate-sampler device. All processed plates were measured on the same day.

Knockdowns

Following ON-TARGETplus SMARTpool siRNAs from Dharmacon were used: human ATM (#L-003201-00), human HDAC1 (#L-003493-00), human mTOR (#L-003008-00), human PIK3CA (#L-003018-00) and a non-targeting siRNA control pool (#D-001810-10). siRNA transfection experiments were carried out using Lipofectamine RNAiMax transfection reagent (Life Technologies) according to the previous report(Rosner et al., 2010). hAFSCs were seeded in 6-well plates at 10-20 % confluence and transfected 12 hours later. Dense hESCs/hiPSCs were split at a 1:20 ratio, seeded on Matrigel-coated 6-well plates and transfected 12 hours later. Cells were harvested between 36 and 96 hours post transfection and analyzed by FACS.

FACS analysis

Single cell suspensions were fixed and permeabilized using Foxp3 Fixation/Permeabilization kit (eBioscience, #00-5521-00) as appropriate. For cell cycle assessments, DNA was stained with DAPI (Sigma, D9542-5MG). Living cells were discriminated using Anti-Ki67-Vio515 human and mouse (Miltenyi, 130-108-830). Apoptotic cells were stained using Brilliant-Violet (BV650) rabbit Anti-active caspase-3 from BD Biosciences (#564096). Intracellular stainings were performed using following directly labeled antibodies: anti-human pS6 S240-APC (Miltenyi, 130-106-562), anti-human and mouse AKT p473-PE (Miltenyi, 130-105-250), anti-human and mouse Oct4A-PE (Miltenyi, 130-105-606), anti-human Pax6-APC (Miltenyi, 130-107-829), anti-ATM phospho-S1981 (Biolegend, #651203), rabbit mAb to HDAC1-AlexaFluor488 (abcam, ab192469). Following extracellular antibodies were used

before fixation of cells: anti-human CD140a (PDGF1Ra)-PE/Cy7 (Biolegend, 323507), anti-human CD184 (CXCR4)-APC/Cy7 (Biolegend, 306527) and anti-human Notch1-APC (Biolegend, 352108). Antibodies were used at dilutions between 1:20 and 1:500. Cells were analyzed on a Cytoflex (Beckman Coulter) equipped with four lasers. Inhibitor-treatments and titrations were performed in a 96-well format and analyzed using the optional 96-well plate loader. Up to seven multicolor combinations have been used simultaneously. Corresponding data was analyzed using FlowJo v10.3 software.

Western Blots

Cells were harvested and prepared as previously described[3]. 10-15 µg (hESCs/hiPSCs) or 20-25 µg of total protein were separated on 8 % PAA gels, wet-transferred to NC-membranes and immunoblotted using following primary and secondary antibodies (all Cell Signaling): pan-AKT (40D4) Mouse mAb (#2920), phospho-AKT (Ser473) (D9E) Rabbit mAb (#4060), PI3 Kinase p110α (C73F8) Rabbit mAb (#4249), HDAC1 (10E2) Mouse mAb (#5356), mTOR (7C10) Rabbit mAb (#2983), ATM (D2E2) Rabbit mAb (#2873), α-tubulin (DM1A) Mouse mAb (Calbiochem, # CP06), anti-rabbit IgG, HRP-linked heavy and light chain antibody (Cell Signaling, #7074) and anti-mouse IgG, HRP-linked heavy and light chain antibody (Cell Signaling, #7076). Signals were detected by chemiluminescence method (Pierce, #32106). Uncropped western blots are available upon request.

Cell size measurements and inhibitor titrations

Cells were harvested using trypsin/EDTA or Accutase and single cell suspensions subjected to the Casy cell counter (Roche) evaluating cell number (cell count) and overall cell size (fl). Dead cells or debris were excluded from cell count and size

measurements. GDC-0941, PI-103, Trichostatin A, Entinostat and Torin-2 have been purchased from Selleckchem.com. Limited dilutions at indicated concentrations were used to establish dose-response curves to inhibitors. All measurements have been performed in triplicates.

Supplemental References

1. Fuchs, C. *et al.* Tuberin and PRAS40 are anti-apoptotic gatekeepers during early human amniotic fluid stem-cell differentiation. *Human molecular genetics* **21**, 1049-1061, doi:10.1093/hmg/ddr535 (2012).
2. Muellner, M. K. *et al.* A chemical-genetic screen reveals a mechanism of resistance to PI3K inhibitors in cancer. *Nature chemical biology* **7**, 787-793, doi:10.1038/nchembio.695 (2011).
3. Rosner, M., Pham, H. T. T., Moriggl, R. & Hengstschlager, M. Human stem cells alter the invasive properties of somatic cells via paracrine activation of mTORC1. *Nature communications* **8**, 595, doi:10.1038/s41467-017-00661-x (2017).

Kovacic_Fig. S1

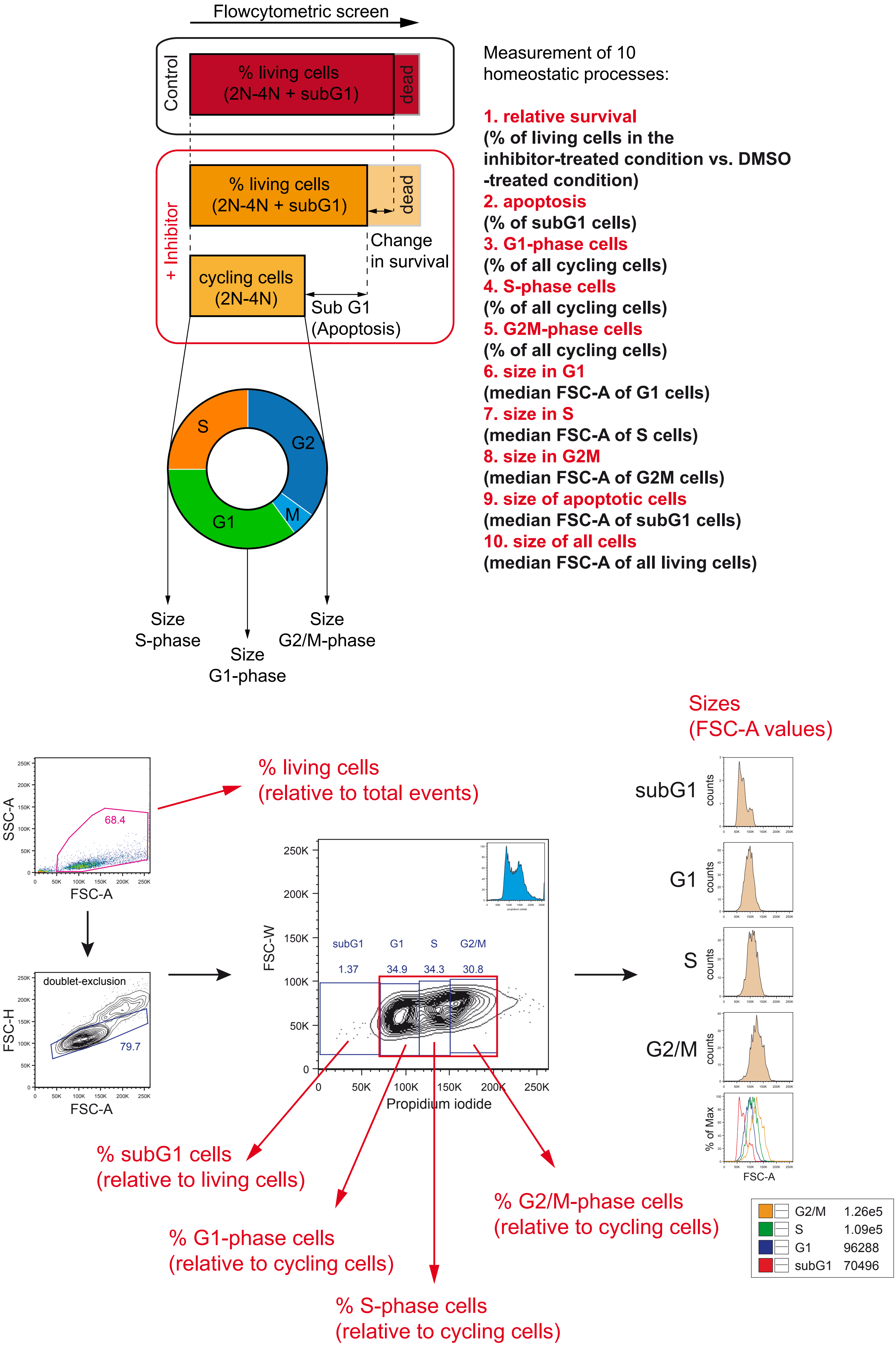

A

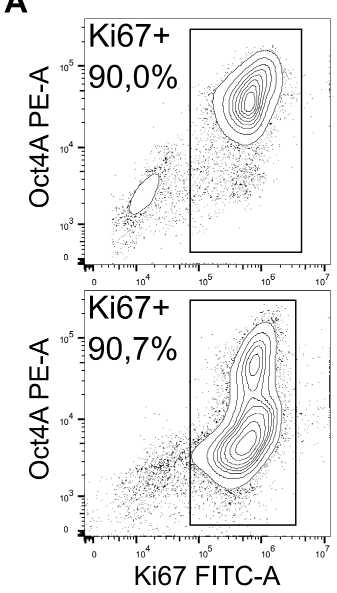

B

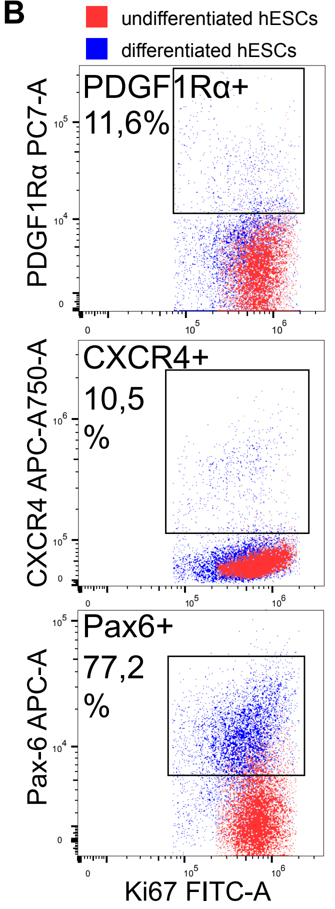

C

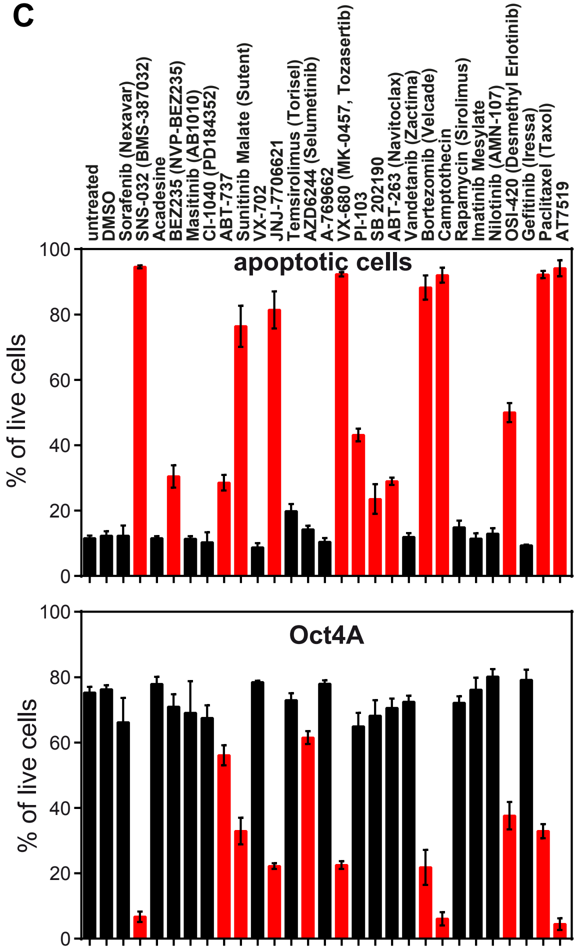

AND / AND / OR / OR

	apoptosis	Oct4A+	Pax6+	CXCR4+	PDGF1Rα+
Control DIFFERENTIATED CELLS	*none*	*reduced*	*increased*	*increased*	*increased*
**Sorafenib (Nexavar)**	*none*	*unchanged*	*increased*	*unchanged*	*unchanged*
**SNS-032 (BMS-387032)**	*increased*	*reduced*	*reduced*	*increased*	*increased*
**Acadesine**	*none*	*unchanged*	*unchanged*	*unchanged*	*unchanged*
**BEZ235 (NVP-BEZ235)**	*increased*	*unchanged*	*unchanged*	*increased*	*increased*
**Masitinib (AB1010)**	*none*	*unchanged*	*unchanged*	*unchanged*	*unchanged*
**CI-1040 (PD184352)**	*none*	*unchanged*	*unchanged*	*unchanged*	*unchanged*
**ABT-737**	*increased*	*reduced*	*increased*	*unchanged*	*unchanged*
**Sunitinib Malate (Sutent)**	*increased*	*reduced*	*reduced*	*increased*	*increased*
**VX-702**	*none*	*unchanged*	*unchanged*	*unchanged*	*unchanged*
**JNJ-7706621**	*increased*	*reduced*	*reduced*	*increased*	*increased*
**Temsirolimus (Torisel)**	*none*	*unchanged*	*unchanged*	*unchanged*	*unchanged*
**AZD6244 (Selumetinib)**	*none*	*reduced*	*unchanged*	*unchanged*	*unchanged*
**A-769662**	*none*	*unchanged*	*unchanged*	*unchanged*	*unchanged*
**VX-680 (MK-0457, Tozasertib)**	*increased*	*reduced*	*reduced*	*increased*	*increased*
**PI-103**	*increased*	*unchanged*	*unchanged*	*increased*	*increased*
**SB 202190**	*increased*	*unchanged*	*unchanged*	*increased*	*unchanged*
**ABT-263 (Navitoclax)**	*increased*	*unchanged*	*unchanged*	*unchanged*	*unchanged*
**Vandetanib (Zactima)**	*none*	*unchanged*	*unchanged*	*unchanged*	*unchanged*
**Bortezomib (Velcade)**	*increased*	*reduced*	*reduced*	*increased*	*increased*
**Camptothecin**	*increased*	*reduced*	*reduced*	*increased*	*increased*
**Rapamycin (Sirolimus)**	*none*	*unchanged*	*unchanged*	*unchanged*	*unchanged*
**Imatinib Mesylate**	*none*	*unchanged*	*unchanged*	*unchanged*	*unchanged*
**Nilotinib (AMN-107)**	*none*	*unchanged*	*unchanged*	*unchanged*	*unchanged*
**OSI-420 (Desmethyl Erlotinib)**	*increased*	*reduced*	*unchanged*	*increased*	*increased*
**Gefitinib (Iressa)**	*none*	*unchanged*	*unchanged*	*unchanged*	*unchanged*
**Paclitaxel (Taxol)**	*increased*	*reduced*	*reduced*	*increased*	*increased*
**AT7519**	*increased*	*reduced*	*reduced*	*increased*	*increased*

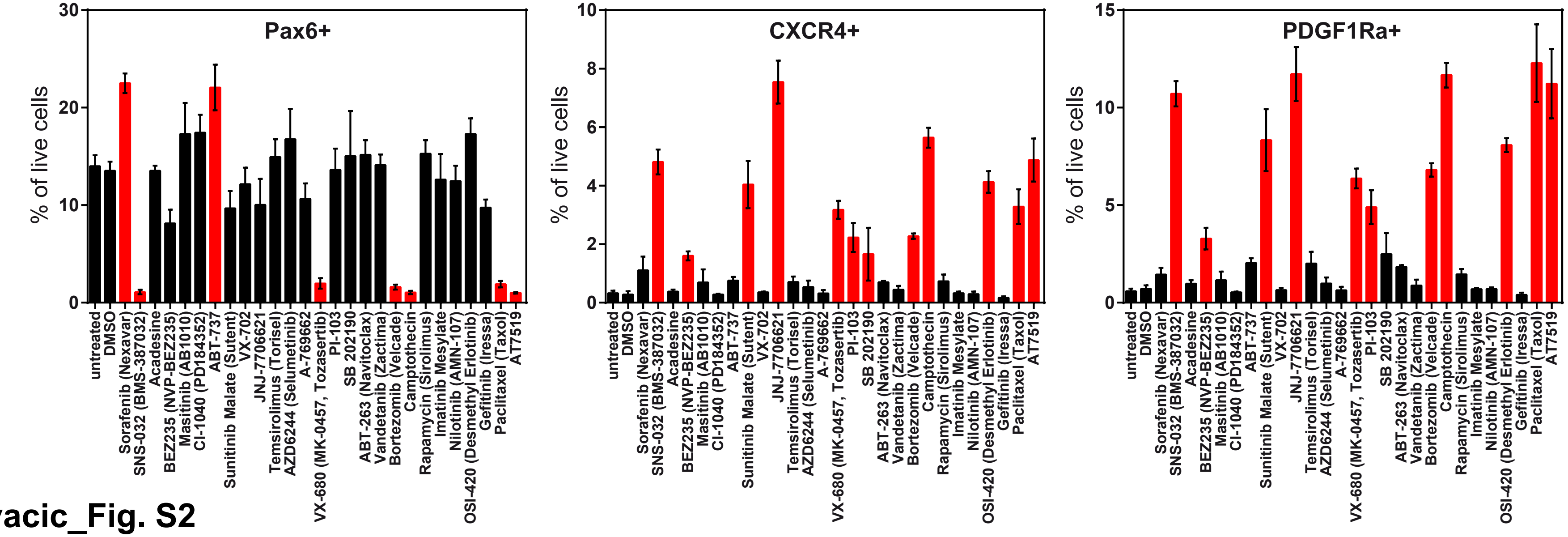

Kovacic_Fig. S2

Kovacic_Fig. S3

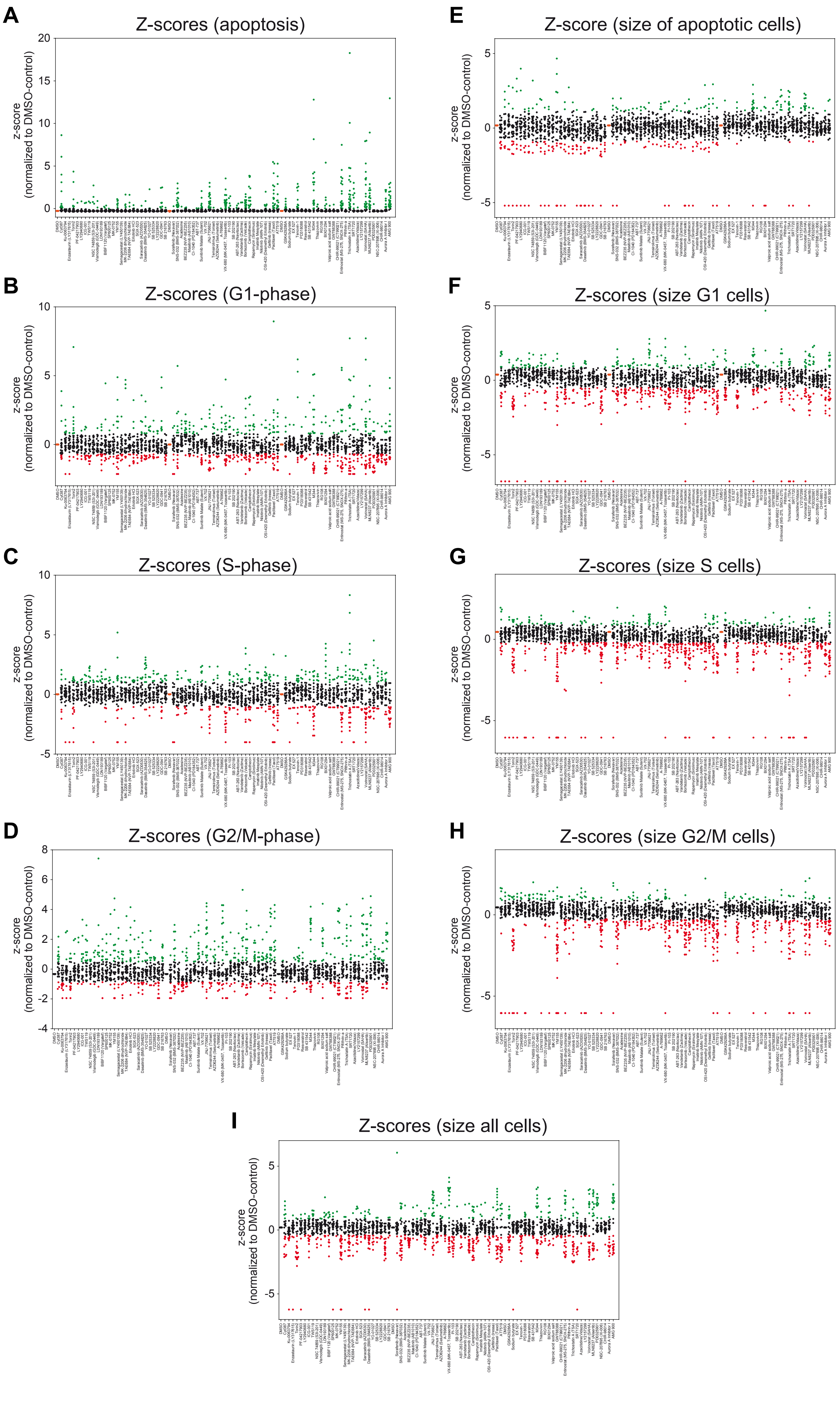

Kovacic_Fig. S4

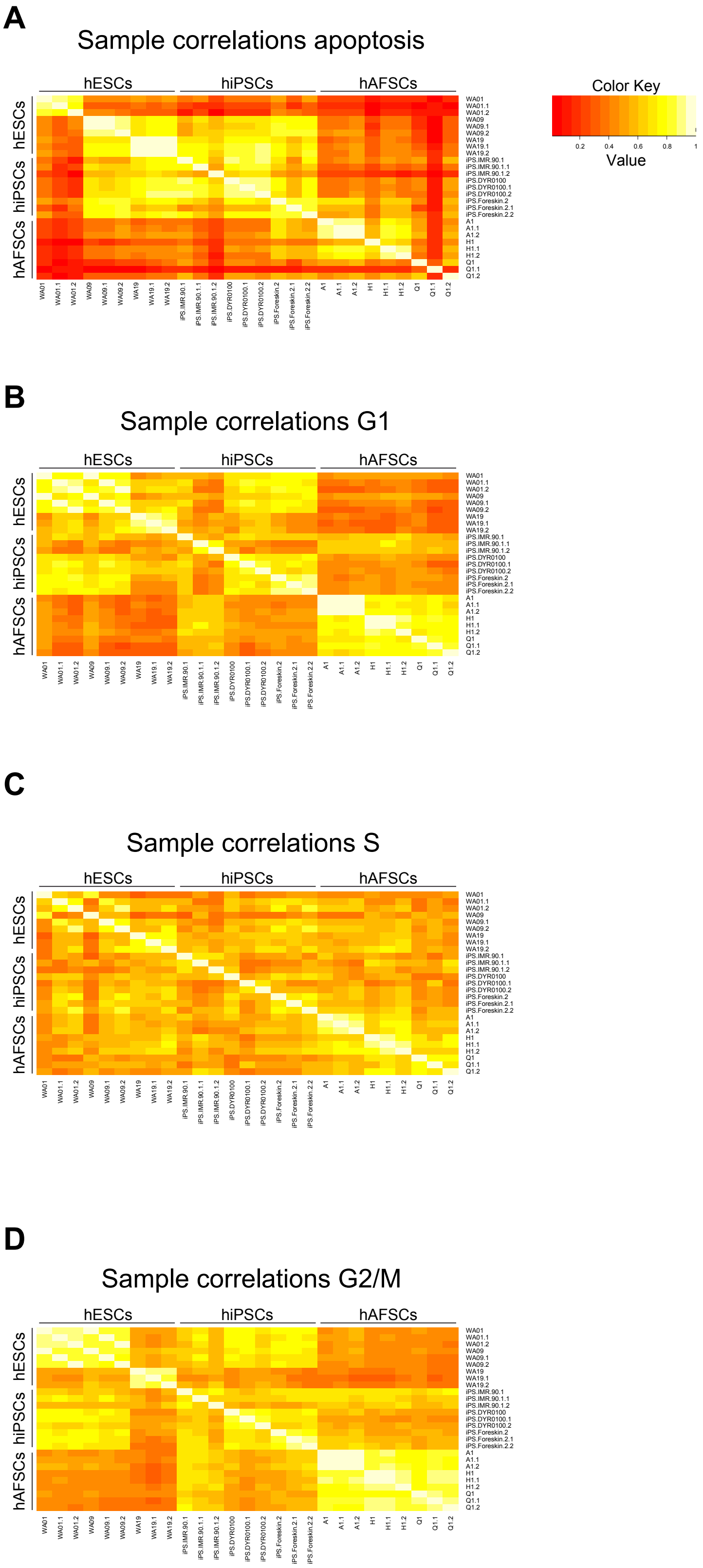

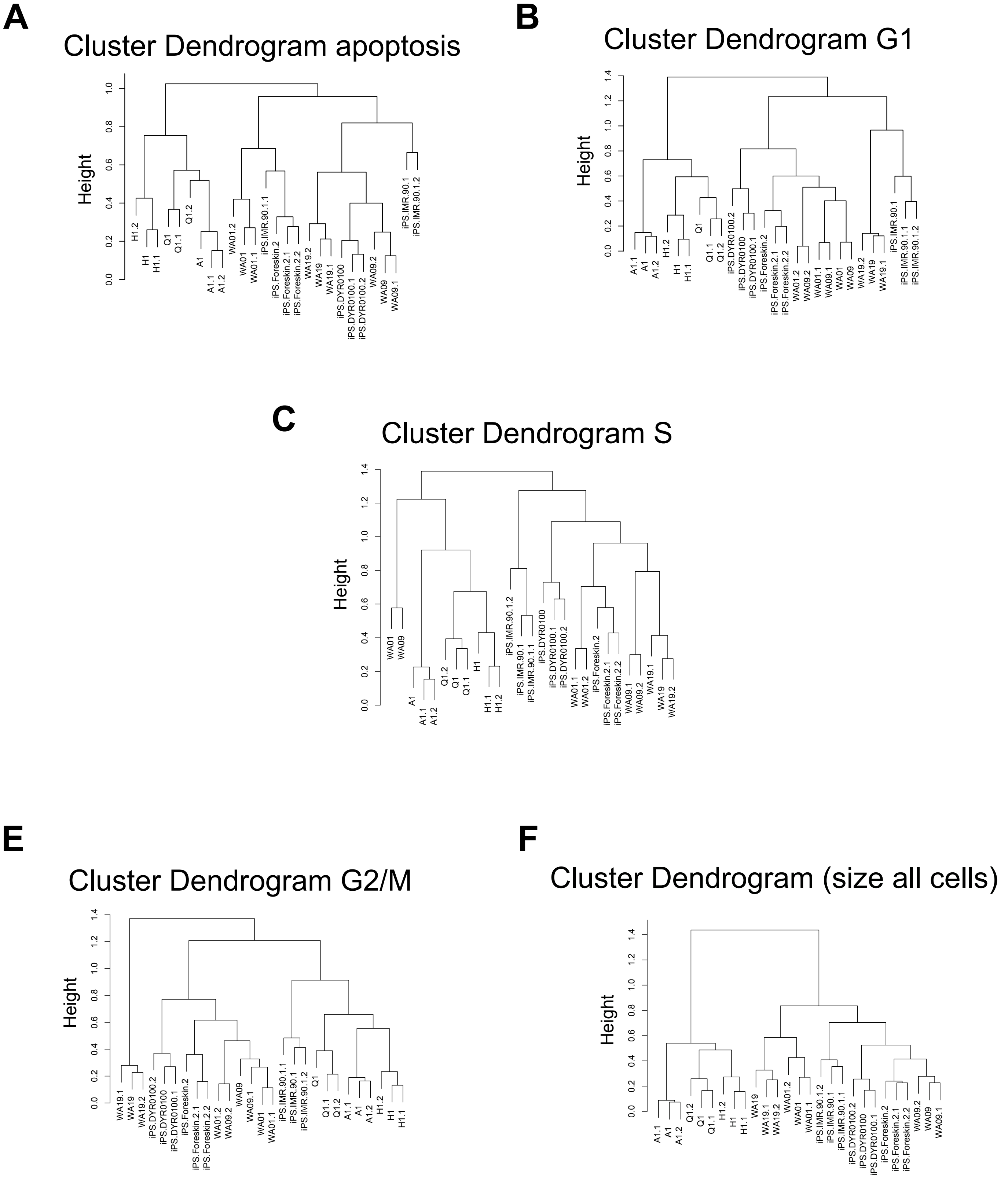
A
Cluster Dendrogram apoptosis
Height
0.0
0.2
0.4
0.6
0.8
1.0
H1.2
H1
H1.1
Q1
Q1.1
Q1.2
A1
A1.1
A1.2
WA01.2
WA01
WA01.1
iPS.IMR.90.1.1
iPS.Foreskin.2
iPS.Foreskin.2.1
iPS.Foreskin.2.2
WA19.2
WA19
WA19.1
iPS.DYR0100
iPS.DYR0100.1
iPS.DYR0100.2
WA09.2
WA09
WA09.1
iPS.IMR.90.1
iPS.IMR.90.1.2
B
Cluster Dendrogram G1
Height
0.0
0.2
0.4
0.6
0.8
1.0
1.2
1.4
A1.1
A1
A1.2
H1.2
H1
H1.1
Q1
Q1.1
Q1.2
iPS.DYR0100.2
iPS.DYR0100
iPS.DYR0100.1
iPS.Foreskin.2
iPS.Foreskin.2.1
iPS.Foreskin.2.2
WA01.2
WA09.2
WA01.1
WA09.1
WA01
WA09
WA19.2
WA19
WA19.1
iPS.IMR.90.1
iPS.IMR.90.1.1
iPS.IMR.90.1.2
C
Cluster Dendrogram S
Height
0.0
0.2
0.4
0.6
0.8
1.0
1.2
1.4
WA01
WA09
A1
A1.1
A1.2
Q1.2
Q1
Q1.1
H1
H1.1
H1.2
iPS.IMR.90.1.2
iPS.IMR.90.1
iPS.IMR.90.1.1
iPS.DYR0100
iPS.DYR0100.1
iPS.DYR0100.2
WA01.1
WA01.2
iPS.Foreskin.2
iPS.Foreskin.2.1
iPS.Foreskin.2.2
WA09.1
WA09.2
WA19.1
WA19
WA19.2
E
Cluster Dendrogram G2/M
Height
0.0
0.2
0.4
0.6
0.8
1.0
1.2
1.4
WA19.1
WA19
WA19.2
iPS.DYR0100.2
iPS.DYR0100
iPS.DYR0100.1
iPS.Foreskin.2
iPS.Foreskin.2.1
iPS.Foreskin.2.2
WA01.2
WA09.2
WA09
WA09.1
WA01
WA01.1
iPS.IMR.90.1.1
iPS.IMR.90.1
iPS.IMR.90.1.2
Q1
Q1.1
Q1.2
A1.1
A1
A1.2
H1.2
H1
H1.1
F
Cluster Dendrogram (size all cells)
Height
0.0
0.2
0.4
0.6
0.8
1.0
1.2
1.4
A1.1
A1
A1.2
Q1.2
Q1
Q1.1
H1.2
H1
H1.1
WA19
WA19.1
WA19.2
WA01.2
WA01
WA01.1
iPS.IMR.90.1.2
iPS.IMR.90.1
iPS.IMR.90.1.1
iPS.DYR0100.2
iPS.DYR0100
iPS.DYR0100.1
iPS.Foreskin.2
iPS.Foreskin.2.1
iPS.Foreskin.2.2
WA09.2
WA09
WA09.1

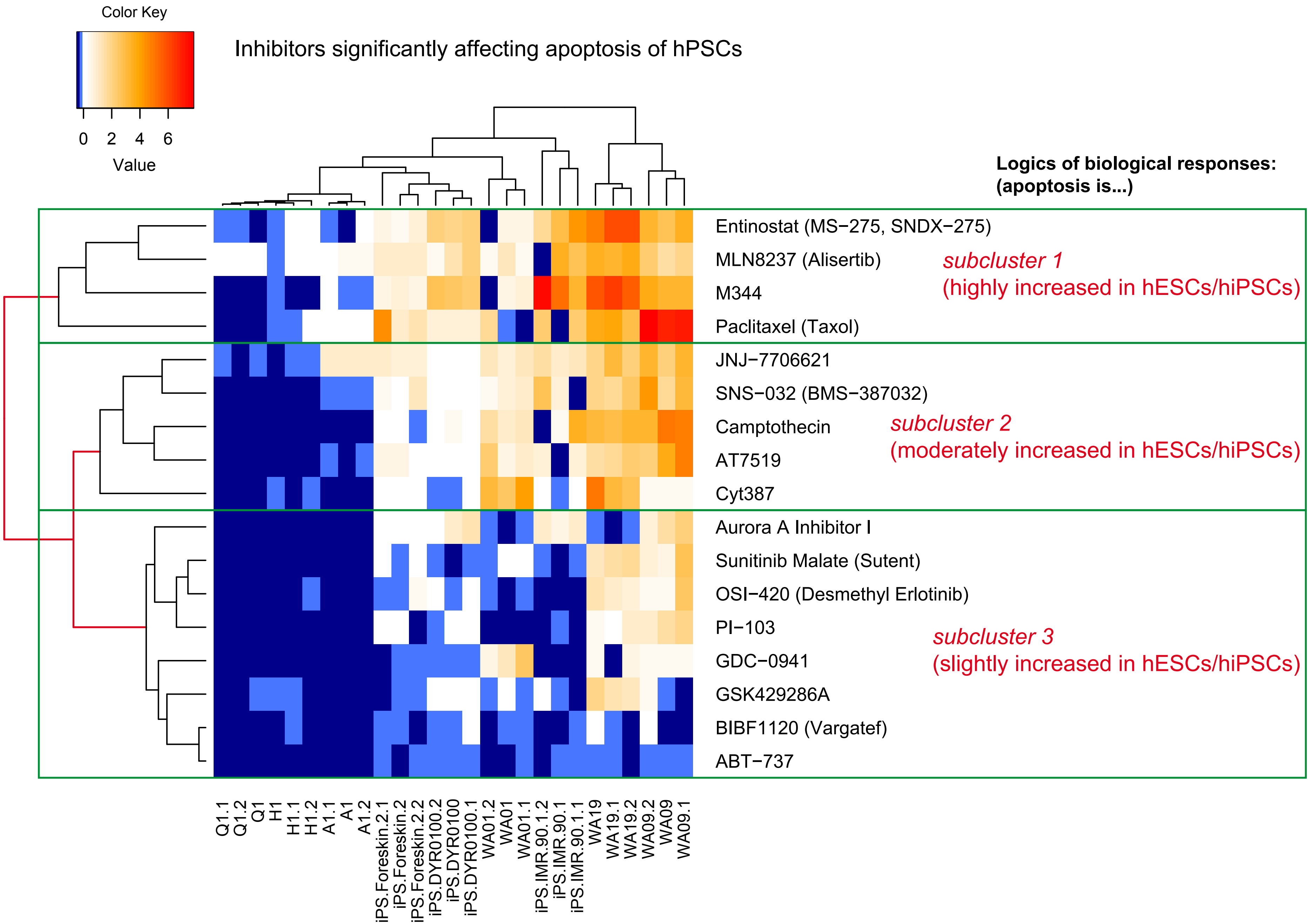
Color Key
0 2 4 6
Value
Inhibitors significantly affecting apoptosis of hPSCs
Logics of biological responses:
(apoptosis is...)
Entinostat (MS−275, SNDX−275)
MLN8237 (Alisertib)
M344
Paclitaxel (Taxol)
subcluster 1
(highly increased in hESCs/hiPSCs)
JNJ−7706621
SNS−032 (BMS−387032)
Camptothecin
AT7519
Cyt387
subcluster 2
(moderately increased in hESCs/hiPSCs)
Aurora A Inhibitor I
Sunitinib Malate (Sutent)
OSI−420 (Desmethyl Erlotinib)
PI−103
GDC−0941
GSK429286A
BIBF1120 (Vargatef)
ABT−737
subcluster 3
(slightly increased in hESCs/hiPSCs)
Q1.1
Q1.2
Q1
H1
H1.1
H1.2
A1.1
A1
A1.2
iPS.Foreskin.2.1
iPS.Foreskin.2
iPS.Foreskin.2.2
iPS.DYR0100.2
iPS.DYR0100
iPS.DYR0100.1
WA01.2
WA01
WA01.1
iPS.IMR.90.1.2
iPS.IMR.90.1
iPS.IMR.90.1.1
WA19
WA19.1
WA19.2
WA09.2
WA09
WA09.1

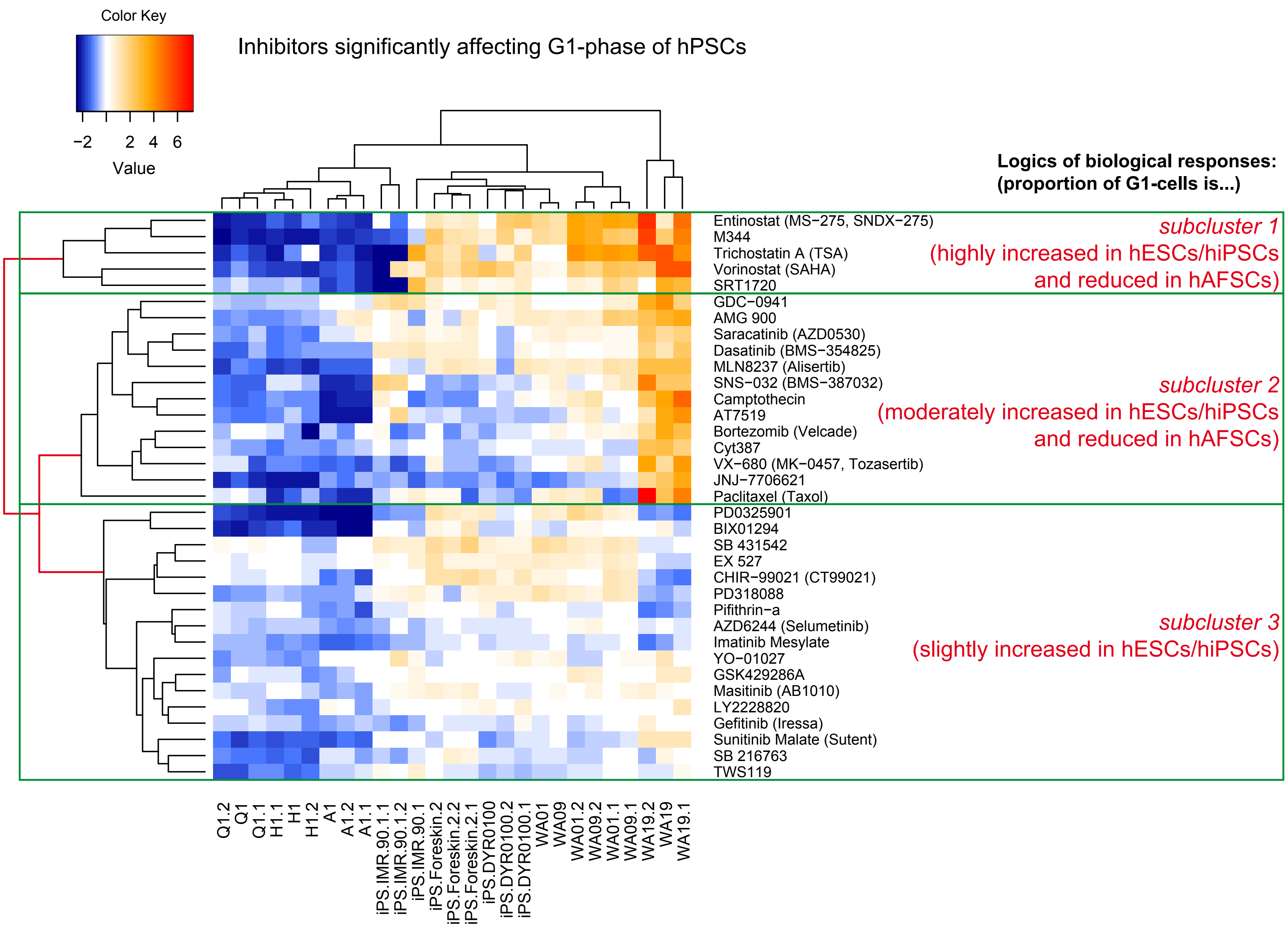
Color Key
−2
2
4
6
Value
Inhibitors significantly affecting G1-phase of hPSCs
Logics of biological responses:
(proportion of G1-cells is...)
Entinostat (MS−275, SNDX−275)
M344
Trichostatin A (TSA)
Vorinostat (SAHA)
SRT1720
GDC−0941
AMG 900
Saracatinib (AZD0530)
Dasatinib (BMS−354825)
MLN8237 (Alisertib)
SNS−032 (BMS−387032)
Camptothecin
AT7519
Bortezomib (Velcade)
Cyt387
VX−680 (MK−0457, Tozasertib)
JNJ−7706621
Paclitaxel (Taxol)
PD0325901
BIX01294
SB 431542
EX 527
CHIR−99021 (CT99021)
PD318088
Pifithrin−a
AZD6244 (Selumetinib)
Imatinib Mesylate
YO−01027
GSK429286A
Masitinib (AB1010)
LY2228820
Gefitinib (Iressa)
Sunitinib Malate (Sutent)
SB 216763
TWS119
subcluster 1
(highly increased in hESCs/hiPSCs
and reduced in hAFSCs)
subcluster 2
(moderately increased in hESCs/hiPSCs
and reduced in hAFSCs)
subcluster 3
(slightly increased in hESCs/hiPSCs)
Q1.2
Q1
Q1.1
H1.1
H1
H1.2
A1
A1.2
A1.1
iPS.IMR.90.1.1
iPS.IMR.90.1.2
iPS.IMR.90.1
iPS.Foreskin.2
iPS.Foreskin.2.2
iPS.Foreskin.2.1
iPS.DYR0100
iPS.DYR0100.2
iPS.DYR0100.1
WA01
WA09
WA01.2
WA09.2
WA01.1
WA09.1
WA19.2
WA19
WA19.1

Kovacic_Fig. S8

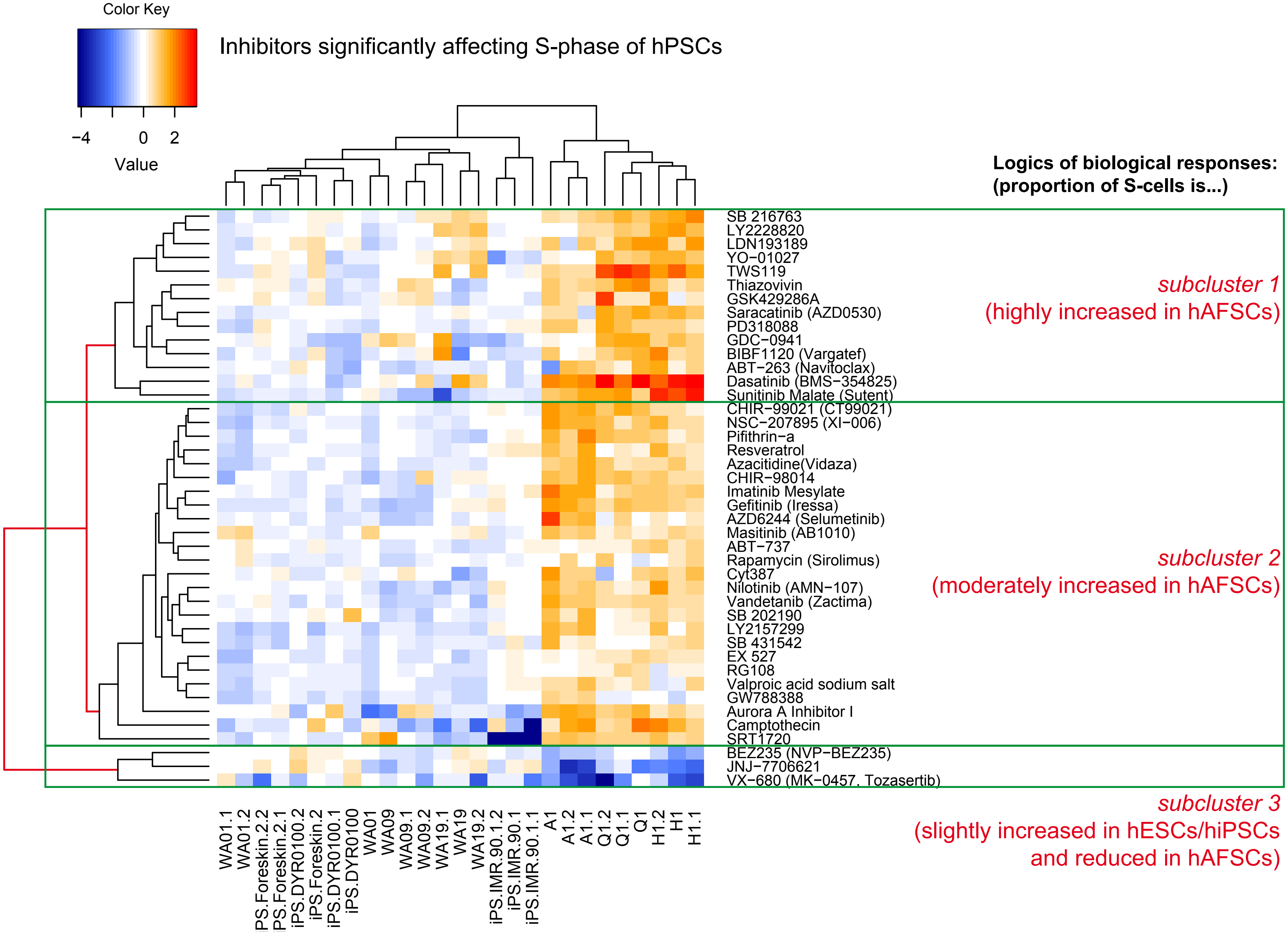

Kovacic_Fig. S9

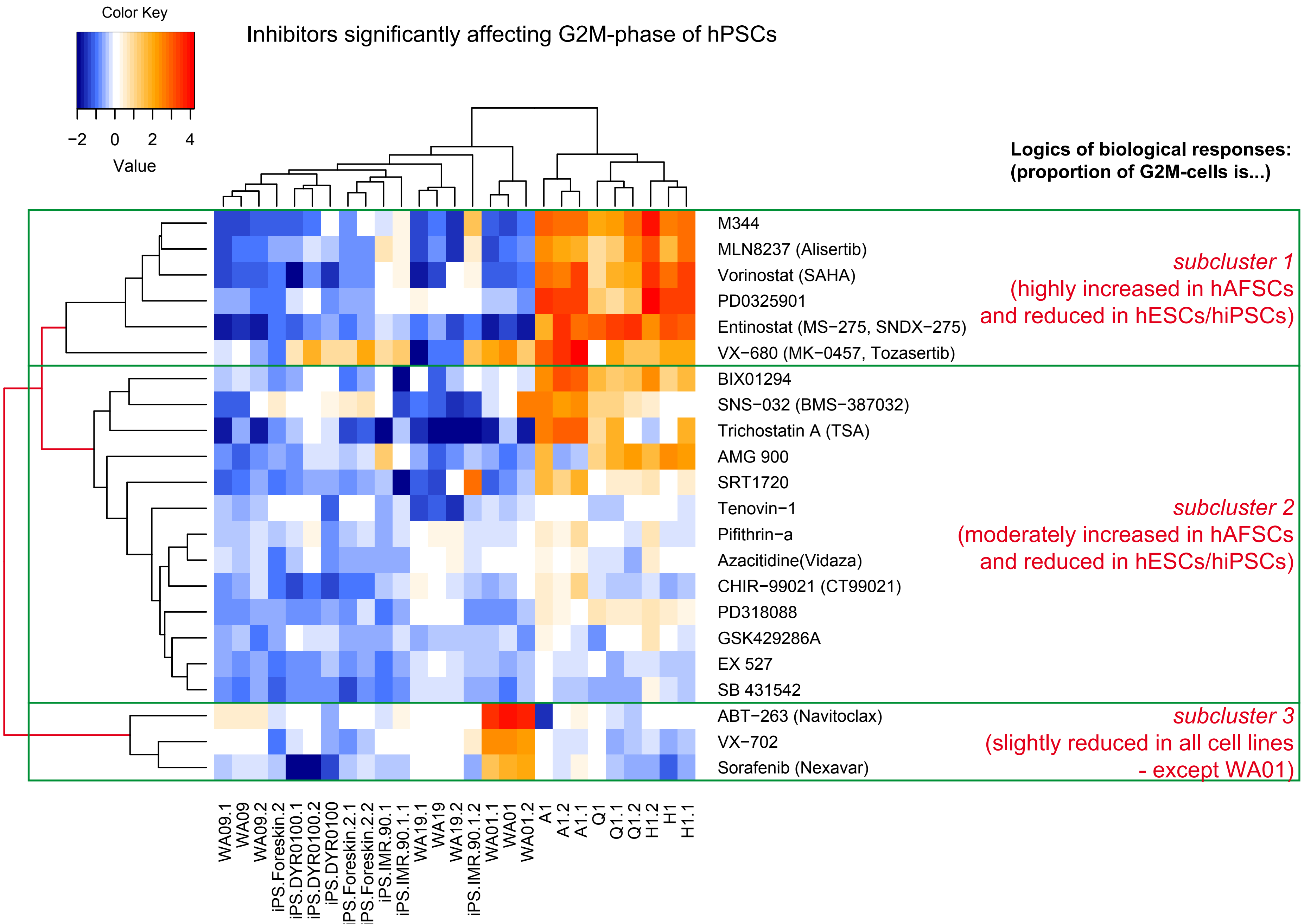

Kovacic_Fig. S10

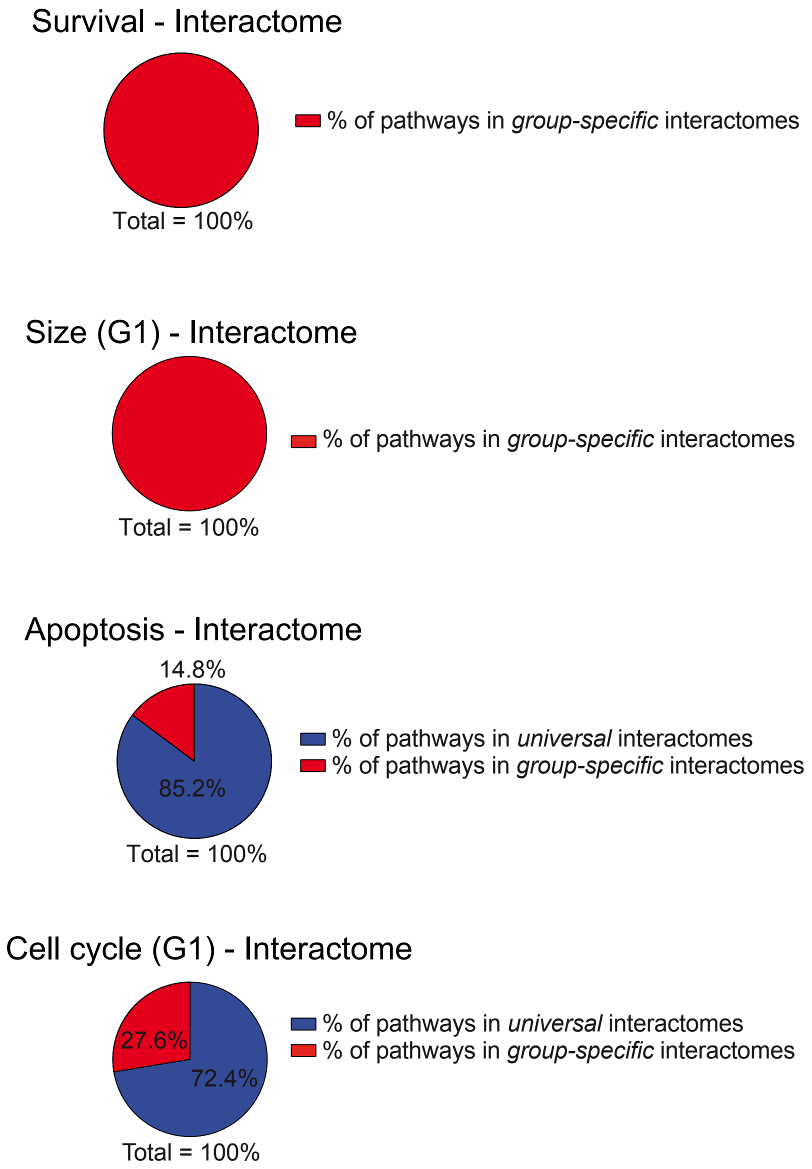

Pathway interaction landscape (S-phase)

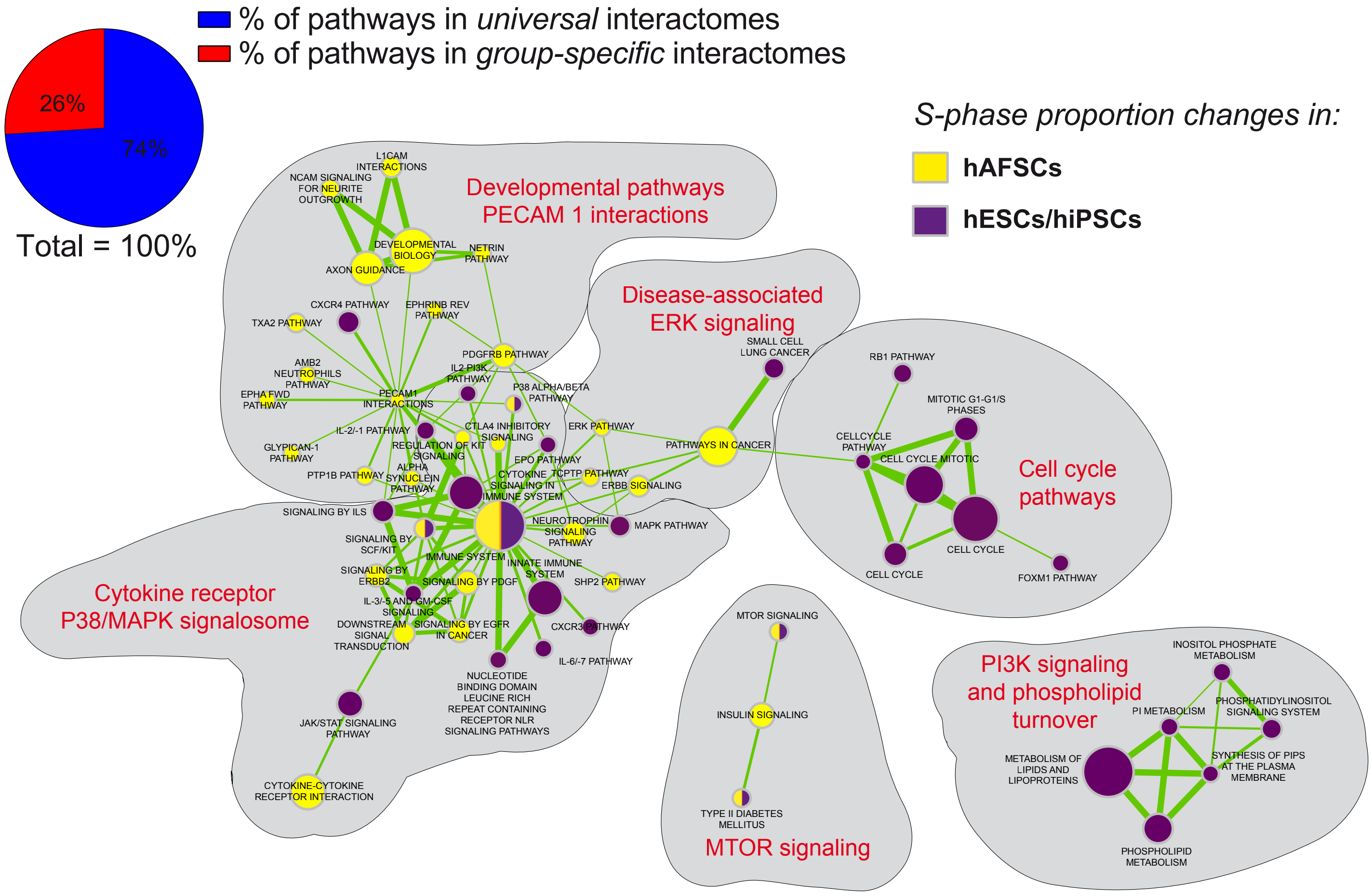

Kovacic_Fig. S12

Pathway interaction landscape of G2/M-phase

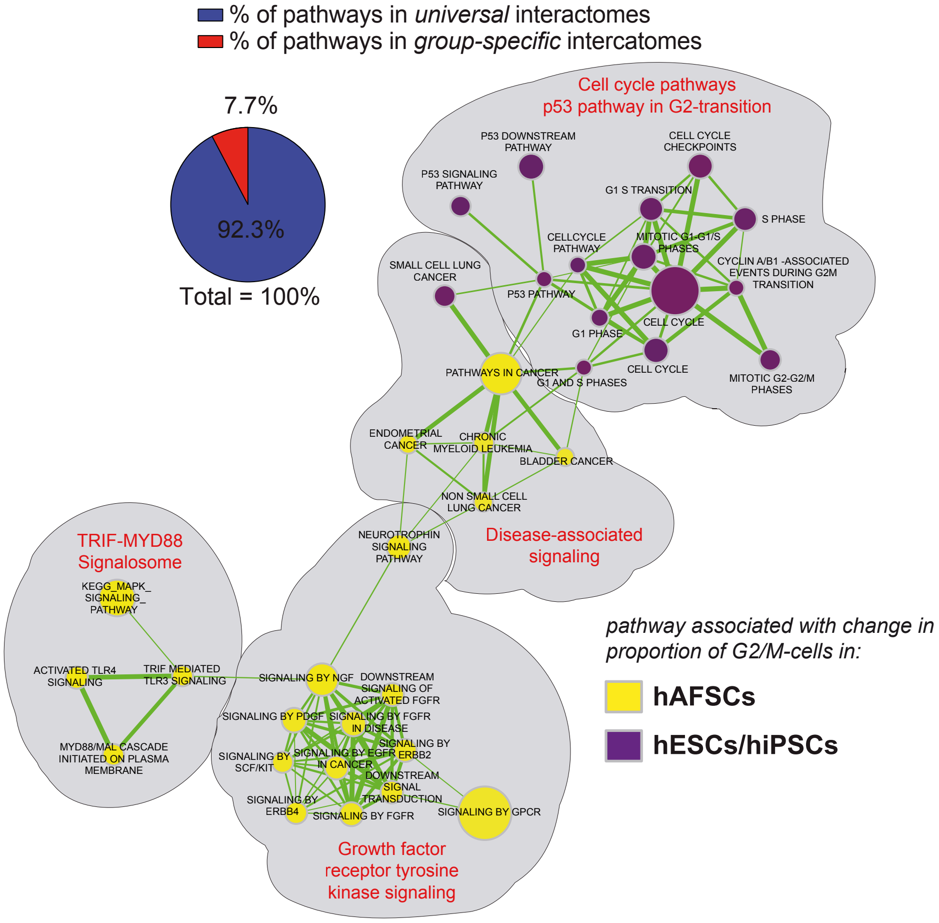

Kovacic_Fig. S13

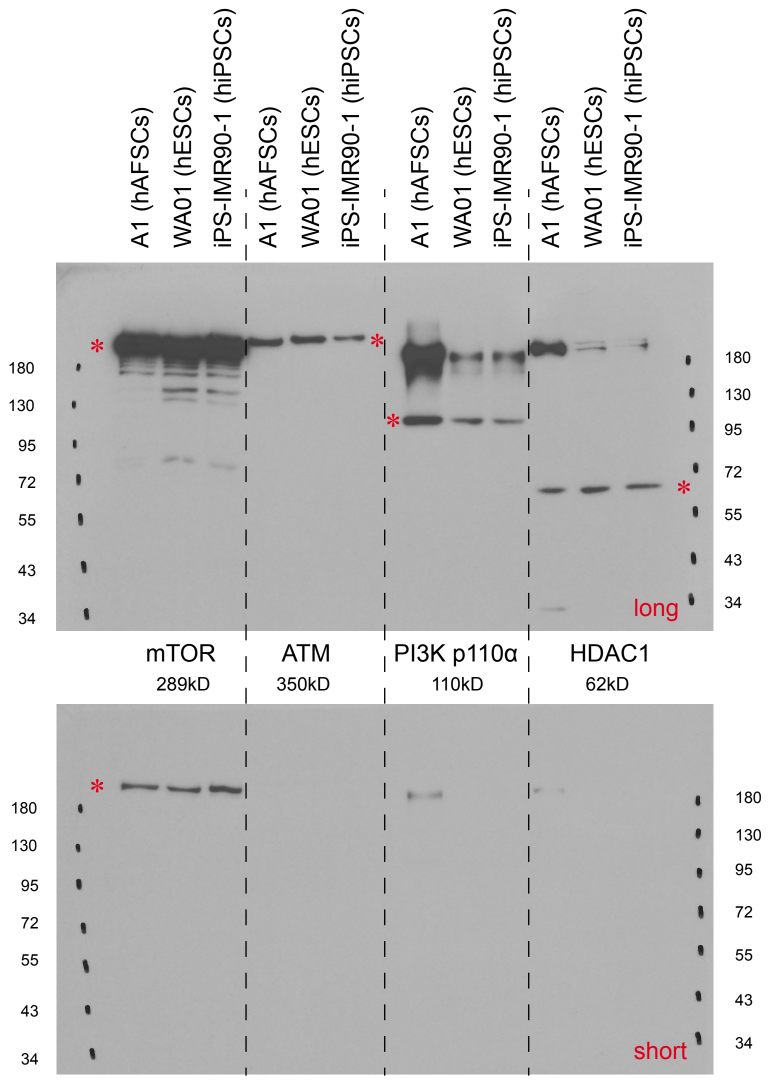

Kovacic_Fig. S14

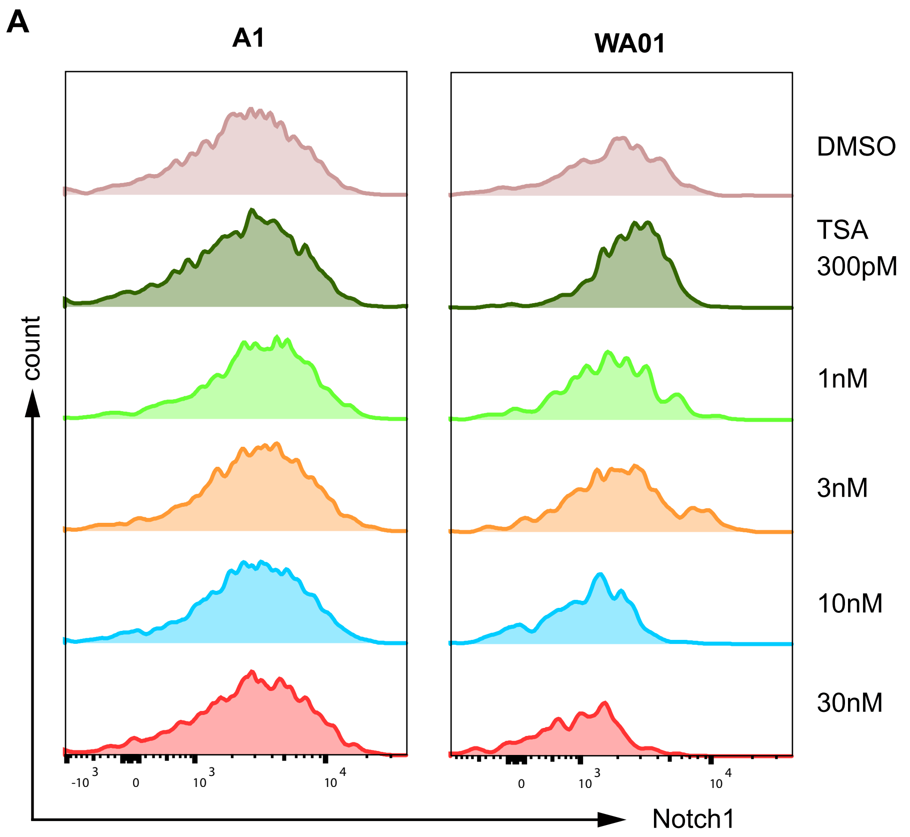

Kovacic_Fig. S15

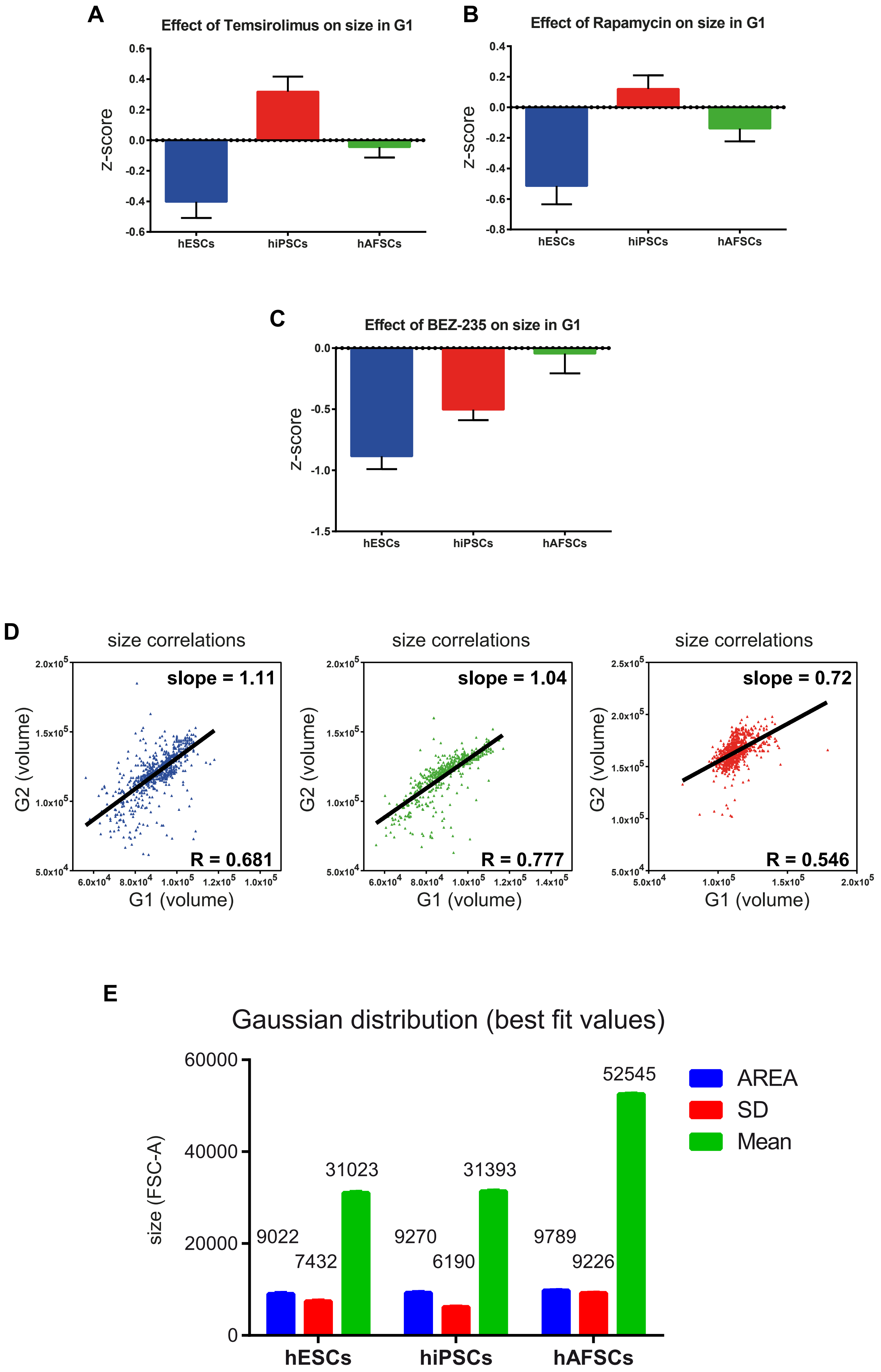

Kovacic_Fig. S16

FDR<0.01

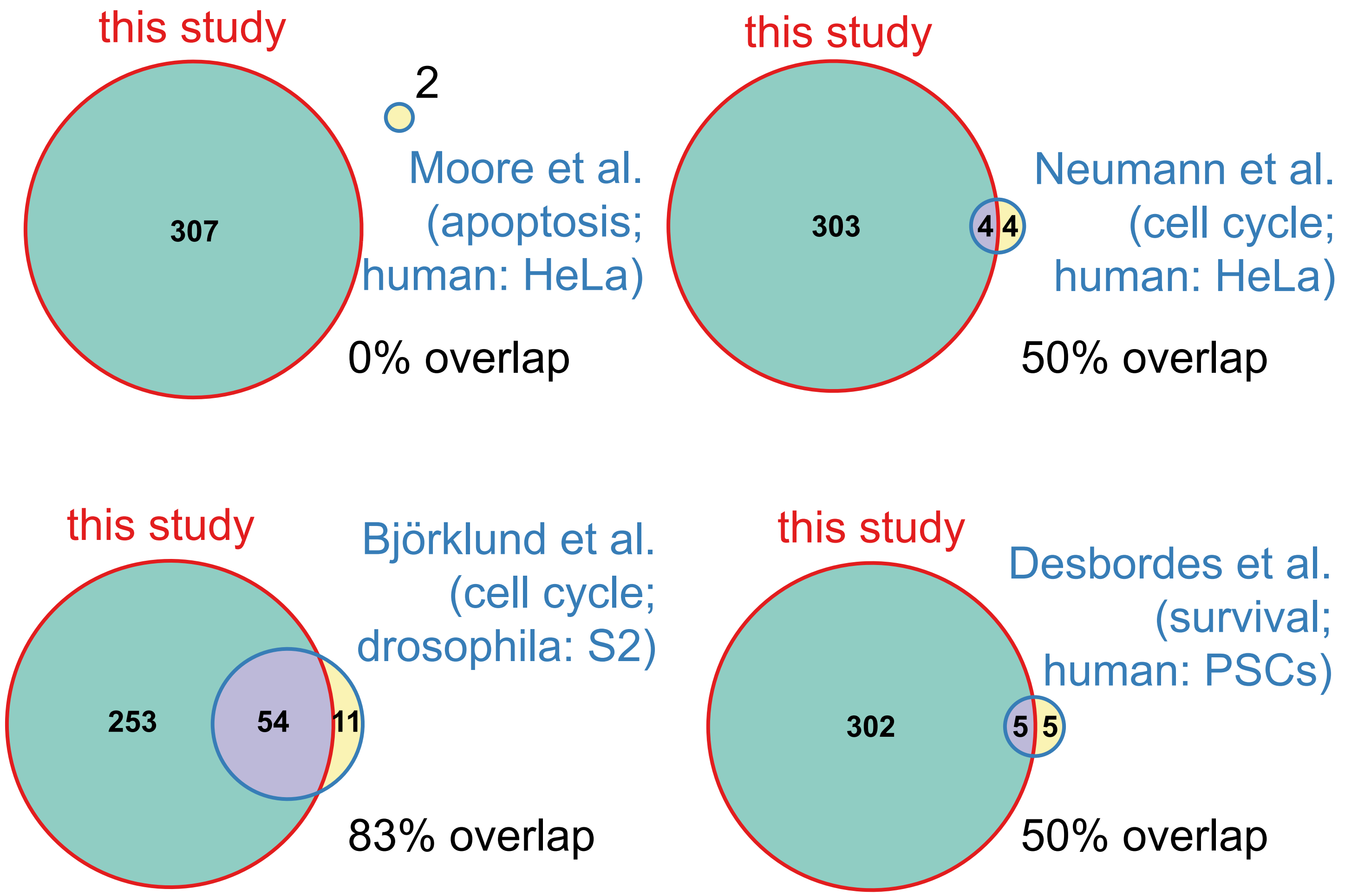

Figure 5A

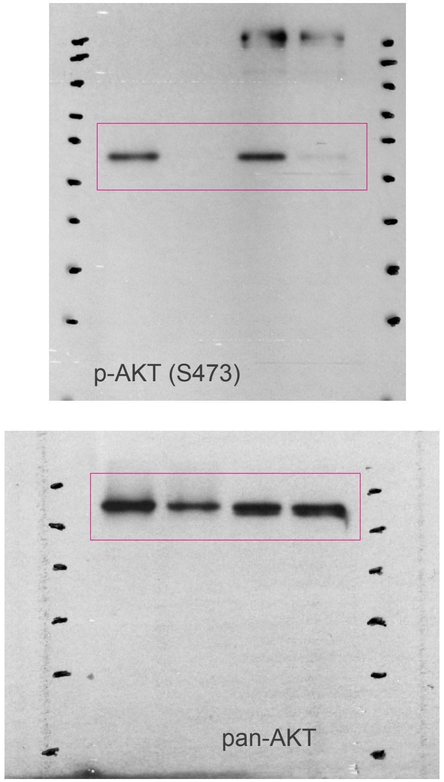

Figure 5D

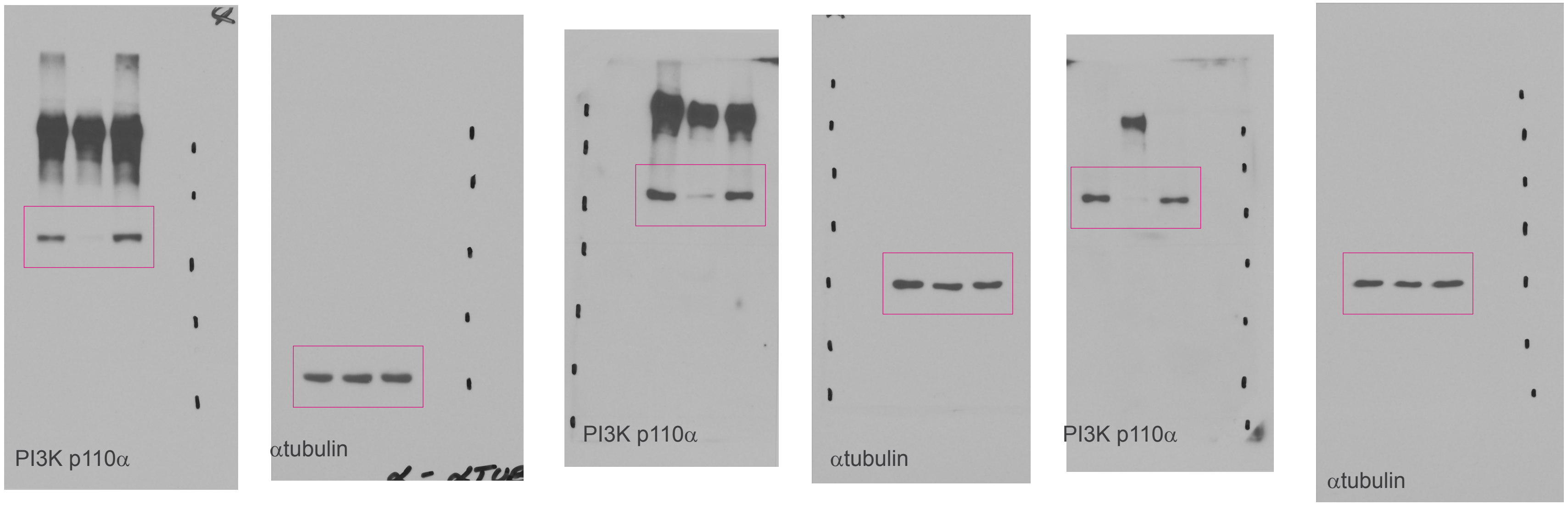

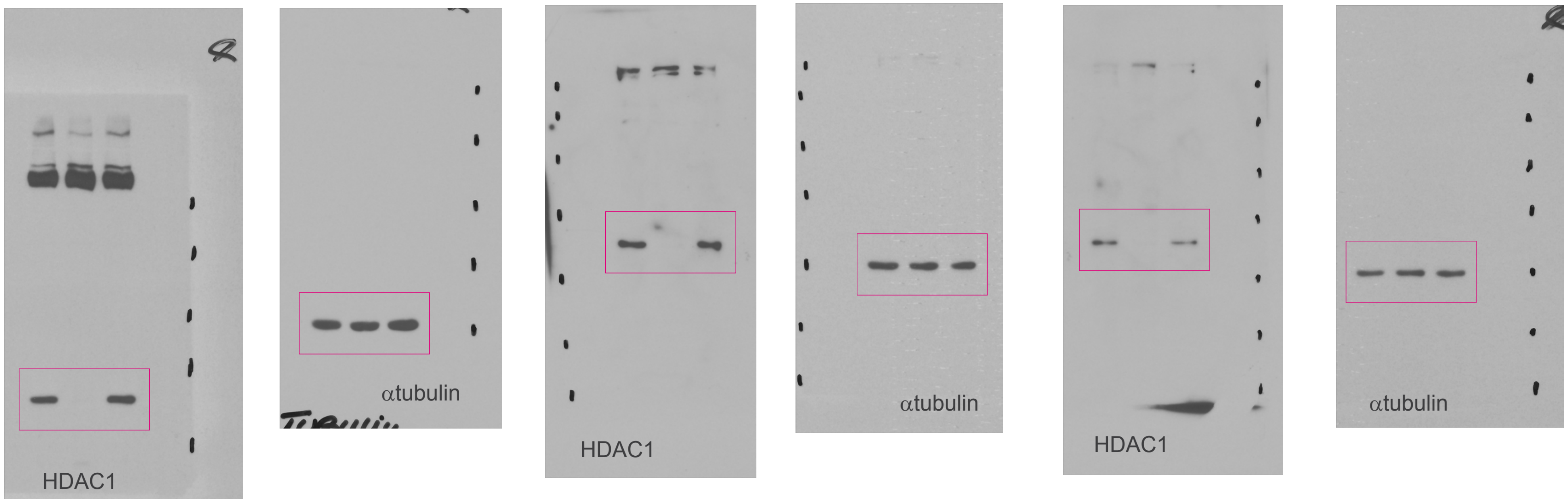

Figure 5K

Figure 6D

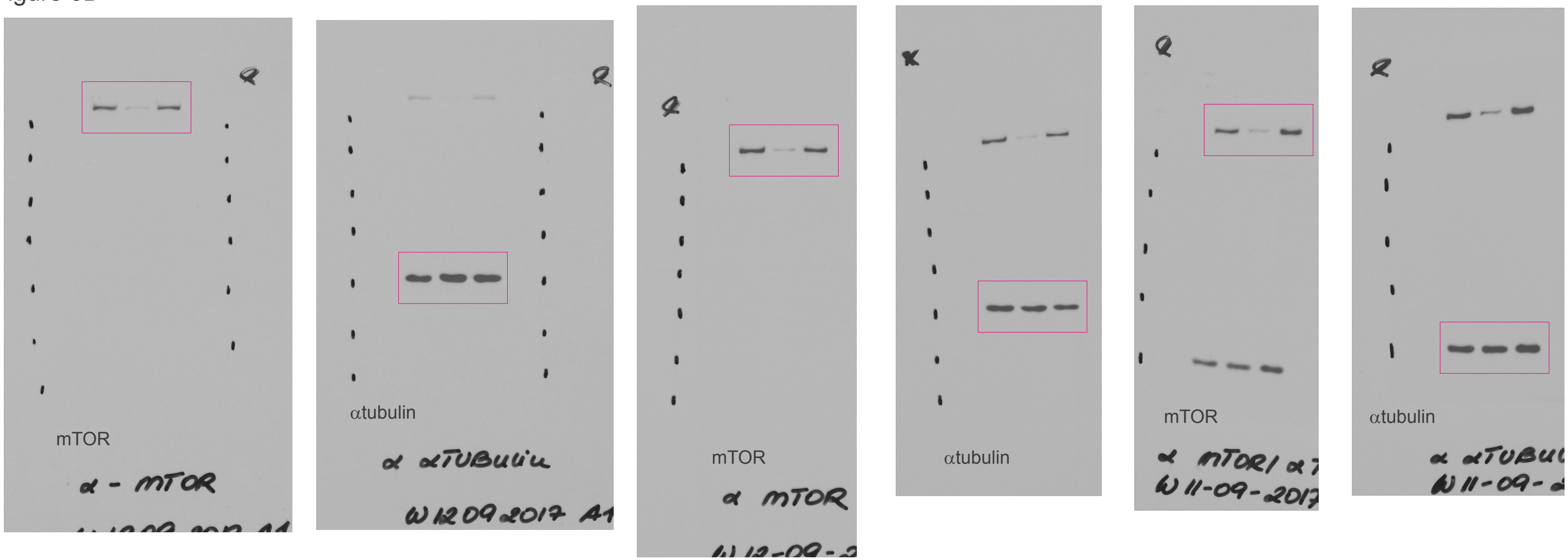

Figure 6F

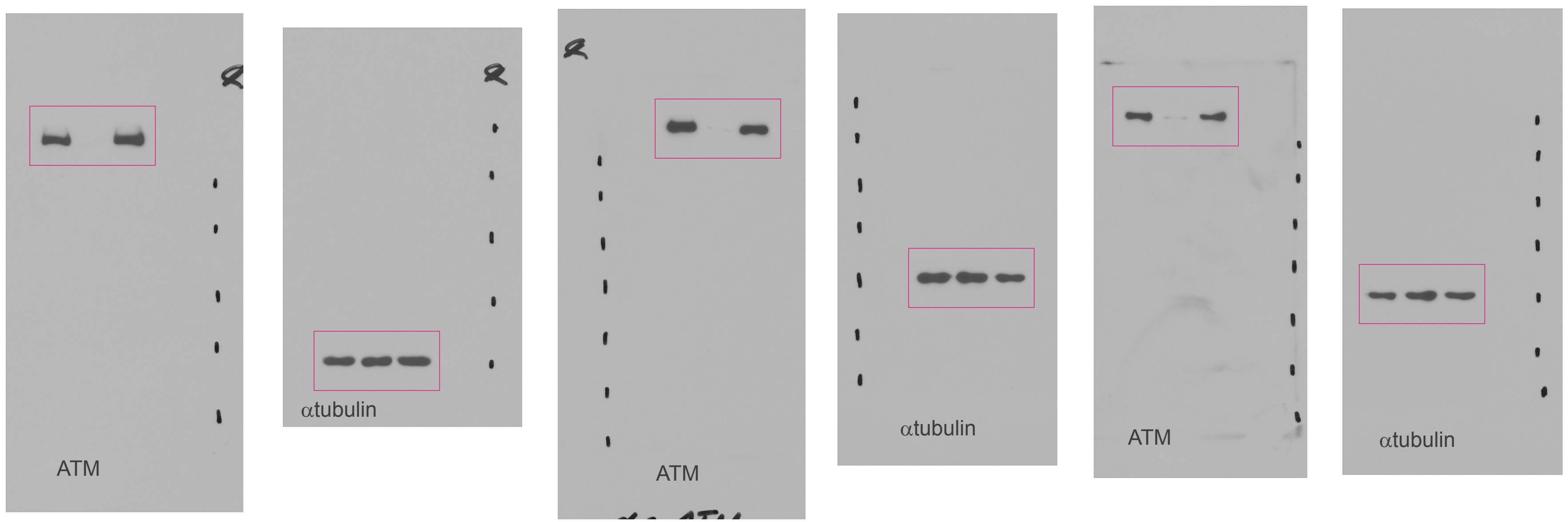

Kovacic_Fig. S18

Supplementary Figure Legends

Figure S1 Schematic of the gating strategy applied to define values accounting for survival, apoptosis, cell cycle phases and their corresponding sizes. To visualize the relative cell survival, we measured the percentage of 'living cells' (live gate) in each condition (controls and inhibitor-treatments). Notably, the variance of the measured percentages was constantly low. PI-stainings have been displayed only from 'living cells' – thus subG1 cells represent only 'early and mid-apoptotic cells' that have just begun the DNA-fragmentation program. Late apoptotic cells are in the 'debris gate' and hence have been excluded from 'apoptotic cells'. All cell cycle stages have been calculated only from 'cycling cells' so that their sum always equals hundred percent.

Figure S2 Pilot-screen to address putative differentiation effects by inhibitors. (A) FACS-plots representing Oct4A and Ki67 stains of undifferentiated (upper panel) and differentiated WA09 cells (lower panel). While Oct4A-expression drops during differentiation, the percentage of Ki67+ cells is stable, suggesting that under regular differentiation conditions, no apoptosis occurs. (B) Blue color - on day 5 after induction of differentiation, the markers for ectoderm (Pax6), endoderm (CXCR4) and mesoderm (PDGF1Rα) appear. Undifferentiated control cells are depicted in red color. (C) Pilot screen - effects of 27 inhibitors on differentiation has been measured after 48 h. Significant changes compared to controls are indicated by red color ($p < 0.05$). The table summarizes results depicted in the bar diagrams. None of the 27 inhibitors has caused a differentiation effect as defined by NO apoptosis, reduced Oct4A expression and increase in expression of either Pax6 or CXCR4 or PDGF1Rα.

Figure S3 Scatterplots of z'-scores from distinct biological processes. (A) 'apoptosis' condition. (B) 'G1-phase' condition. (C) 'S-phase' condition. (D) 'G2/M-phase' condition. (E) 'size of apoptotic cells' condition. (F) 'size of G1 cells' condition. (G) 'size S cells' condition. (H) 'size G2/M cells' condition. (I) 'size all cells' condition. Significant hits have been determined by student's t-test of z'-scores. Values above or beneath the noise-cutoff represent significant hits; $p < 0.01$.

Figure S4 Heatmaps showing Pearson correlations between all samples in distinct biological processes. (A) 'apoptosis', (B) 'G1-phase', (C) 'S-phase, (D) 'G2/M-phase' and (E) 'size of all cells'.

Figure S5 Dendrograms depicting Euclidian distance using Spearman correlations between all samples in distinct biological processes. (A) 'apoptosis', (B) 'G1-phase', (C) 'S-phase', (D) 'G2/M-phase' and (E) 'size of all cells'. Note the separate clusters of hAFSCs and the lack of clustering between hESCs and hiPSCs.

Figure S6 Heatmap showing hierarchical clustering for apoptosis. Major subclusters arising by hierarchical clustering are depicted by red boxes.

Figure S7 Heatmap showing hierarchical clustering for the G1-phase. Major subclusters arising by hierarchical clustering are depicted by red boxes.

Figure S8 Heatmap showing hierarchical clustering for the S-phase. Major subclusters arising by hierarchical clustering are depicted by red boxes.

Figure S9 Heatmap showing hierarchical clustering for the G2/M-phase. Major subclusters arising by hierarchical clustering are depicted by red boxes.

Figure S10 Pathways landscapes from Figure 4 have been resolved into universal or group-specific interactomes. Percentages are shown in the pie-charts.

Figure S11 Pathway interaction landscape for the S-phase. Pathways in which the proportion of cells in S-phase changed during inhibitor-treatment in hAFSCs are represented by yellow nodes. Pathways changing the S-phase proportion in hESCs/hiPSCs are colored in lilac. The size of the nodes is proportional to the total number of genes within the pathway, while the thickness of interactions is proportional to the number of shared genes (cutoff: 0.05). The percentage of pathways in group-specific or in universal interactomes is shown in the respective pie-chart. Nodes with dual colors indicate enrichment to all hPSC-groups.

Figure S12 Pathway interaction landscape for the G2M-phase. Pathways in which the proportion of cells in G2M-phase changed during inhibitor-treatment in hAFSCs are represented by yellow nodes. Pathways changing the S-phase proportion in

hESCs/hiPSCs are colored in lilac. The size of the nodes is proportional to the total number of genes within the pathway, while the thickness of interactions is proportional to the number of shared genes (cutoff: 0.05). The percentage of pathways in group-specific or in universal interactomes is shown in the respective pie-chart. Nodes with dual colors indicate enrichment to both groups.

Figure S13 A1, WA01 and iPS-IMR90-1 cells express mTOR, ATM, PI3Kα and HDAC1 at comparable levels under homeostatic conditions. Western blots showing mTOR-, ATM-, PI3Kα- and HDAC1-levels in A1, WA01 and iPS-IMR90-1 cells. Specific bands are indicated with red asterisks on long (upper panel) and short (lower panel) exposures. Of note, equal amounts of the same lysates were loaded four times onto a single gel. After transfer, the membrane has been vertically cut, incubated with the respective primary and secondary antibodies, and reassembled for detection.

Figure S14 Molecular effect of TSA-treatments. Notch1 expression levels diminish during G1-phase upon increasing TSA concentrations in WA01, but not in A1 cells.

Figure S15 Predicted differences in size control between hESCs/hiPSCs and hAFSCs are not solely related to mTOR-signaling (A) Average effect of Temsirolimus-treatment on size of distinct hPSC-groups. (B) Average effect of Rapamycin on size of hPSC-groups. (C) Average effect of BEZ-235 on size of distinct hPSC-groups. Of note, size effects by these inhibitors seem not to be in accordance with clusters arising by sample correlations. (D) Volume correlations of median FSC-

A values measured in G1- versus G2-phases. All hPSCs increase size as they progress through cell cycle. Slopes and correlation coefficients are presented for each graph. (E) Gaussian Distribution (best fit values) of all average sizes in G1 using all conditions in the screen. hAFSCs exert the highest mean size and standard deviation (SD).

Figure S16 Analysis of previously reported data with the pathway interactome method. Venn's diagrams of pathways identified in this study and previously reported screens from independent laboratories. Overlapping and non-overlapping pathways are indicated by numbers. Overall, only a limited number of pathways could be retrieved using these data and there was little to no overlap between three studies and our data. However, data obtained by one study yielded > 83% overlap with our data, although only a limited amount of pathways could be retrieved from databases compared to our data.

Figure S17 uncropped western blots from pictures presented in Figures 5A, 5D and 5K.

Figure S18 uncropped western blots from pictures presented in Figures 6D and 6F

Supplementary Table Legends

Table S1

Inhibitors and cell lines used in this study

Table S2

Inhibitors with significant differences among distinct stem cell-groups (n = 3) for each biological process ($p < 0.01$)

Table S3

Inhibitors with no significant effects and without differences among distinct stem cell-groups. Inhibitors with absolute effectivity (“killers”), but no relative difference between hPSC-groups are indicated in yellow.

Table S4

Lists of all previously validated targets (gene names) for all used inhibitors. Information about inhibitor targets have been obtained from www.selleckchem.com

Table S5

Comparisons of Reactome, String and GO (DAVID) pathway analyses in distinct biological processes and different clusters obtained by hierarchical clustering. A maximum of twenty highest-ranked terms has been listed for each process and cluster.

Table S6

Defined subclusters including validated targets (query = gene name) and fold-changes of z-scores for each significant inhibitor and for each biological process

Table S7

GSEA-Identification of significant pathways for hESCs/hiPSCs and hAFSCs in distinct biological processes. The presented lists have been subsequently used for pathway-network construction using the Cytoscape-platform.
